# The Positive Switching Fluorescent Protein Padron2 Enables Live-Cell Reversible Saturable Optical Linear Fluorescence Transitions (RESOLFT) Nanoscopy without Sequential Illumination Steps

**DOI:** 10.1021/acsnano.0c08207

**Published:** 2021-05-21

**Authors:** Timo Konen, Daniel Stumpf, Tim Grotjohann, Isabelle Jansen, Mariano Bossi, Michael Weber, Nickels Jensen, Stefan W. Hell, Stefan Jakobs

**Affiliations:** † Department of NanoBiophotonics, Max Planck Institute for Biophysical Chemistry, 37077 Göttingen, Germany; ‡ Department of Optical Nanoscopy, Max Planck Institute for Medical Research, 69120 Heidelberg, Germany; § Clinic of Neurology, University of Göttingen, 37075 Göttingen, Germany

**Keywords:** super-resolution microscopy, Padron, switching, live cell, fluorescent protein

## Abstract

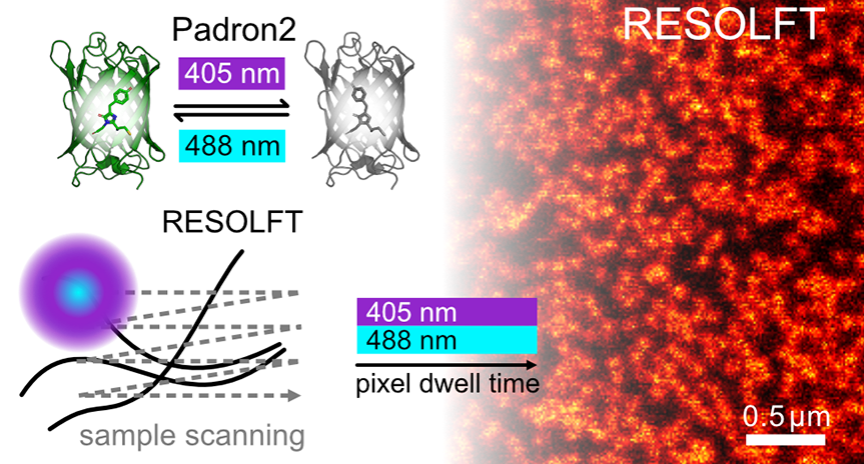

Reversibly switchable fluorescent proteins (RSFPs) can be repeatedly transferred between a fluorescent on- and a nonfluorescent off-state by illumination with light of different wavelengths. Negative switching RSFPs are switched from the on- to the off-state with the same wavelength that also excites fluorescence. Positive switching RSFPs have a reversed light response, where the fluorescence excitation wavelength induces the transition from the off- to the on-state. Reversible saturable optical linear (fluorescence) transitions (RESOLFT) nanoscopy utilizes these switching states to achieve diffraction-unlimited resolution but so far has primarily relied on negative switching RSFPs by using time sequential switching schemes. On the basis of the green fluorescent RSFP Padron, we engineered the positive switching RSFP Padron2. Compared to its predecessor, it can undergo 50-fold more switching cycles while displaying a contrast ratio between the on- and the off-states of more than 100:1. Because of its robust switching behavior, Padron2 supports a RESOLFT imaging scheme that entirely refrains from sequential switching as it only requires beam scanning of two spatially overlaid light distributions. Using Padron2, we demonstrate live-cell RESOLFT nanoscopy without sequential illumination steps.

## Introduction

Nanoscopy, or diffraction-unlimited super-resolution fluorescence microscopy, enables the visualization of cellular structures at the nanoscale. The key to fundamentally overcome the diffraction barrier is to make adjacent molecules discernible through a fluorescence on-/off-state transition forcing nearby fluorophores to emit sequentially.^1^ This separation of fluorophores can be implemented either in a coordinate-targeted or in a coordinate-stochastic way (for reviews see refs (2 and 3)).

RESOLFT (reversible saturable optical linear (fluorescence) transitions) nanoscopy is a coordinate-targeted approach that relies on reversibly switchable fluorophores.^4−7^ In point-scanning RESOLFT nanoscopy, a single laser beam creating a doughnut-shaped intensity distribution with a zero at its center is used to transfer molecules into a nonfluorescent off-state. Thereby, the on-state molecules and hence emission are limited to a central region smaller than the diffraction limit, which is read out by a regularly focused Gaussian beam.

Most implementations of RESOLFT nanoscopy rely on reversibly switchable fluorescent proteins (RSFPs), which belong to the group of GFP-like fluorescent proteins.^8,9^ These proteins feature a β-barrel structure with a central α-helix containing the autocatalytically formed chromophore.^10^ RSFPs can be reversibly toggled between a fluorescent on- and a nonfluorescent off-state by illumination with two different wavelengths. Because RSFPs are metastable in both the on- and the off-state and the quantum yield for switching is comparatively high, the light doses and intensities required for overcoming the diffraction barrier are low compared to basically all other nanoscopy approaches.^11^ In fact, the light intensities used are similar to those applied in live-cell confocal fluorescence microscopy. Because the intensity of illumination and the applied overall light doses are important factors that determine phototoxicity,^12,13^ RESOLFT nanoscopy is particularly suitable for live-cell recordings.

The switching behavior of most RSFPs has been classified as either negative or positive switching.^14^ Negative switching RSFPs are switched from the on- to the off-state with the same wavelength, which is also used for fluorescence excitation. In positive switching RSFPs, the excitation wavelength induces the off-to-on transition. In both classes of RSFPs, the respective other switching direction is triggered by a second, shorter wavelength, typically with a higher switching quantum yield. The molecular basis of this switching process is a cis–trans isomerization of the chromophore, usually accompanied by a protonation change of the chromophore.^8^ In most negative switching RSFPs such as rsEGFP, the on-state is formed by a (largely) deprotonated cis-chromophore and the off-state by a (largely) protonated trans-chromophore.^15,16^ In contrast, in most positive switching RSFPs such as Padron, the off-state is formed by a deprotonated trans-chromophore, while the on-state consists of an equilibrium of protonated and deprotonated cis-chromophores.^17,18^ In the on-state equilibrium, exciting the deprotonated chromophore induces fluorescence, while exciting the protonated chromophore facilitates the transition to the off-state.

At present, almost all RESOLFT implementations rely on RSFPs with a negative switching mode.^9^ In a typical RESOLFT scheme using negative switching RSFPs, fluorophores are switched sequentially.^4,5,19−23^ Thereby, after initial switching to the on-state, RSFPs are switched off with a doughnut-shaped beam or a standing wave light pattern and the remaining on-state fluorophores are probed with a regularly focused beam. Because for negative switching RSFPs fluorescence readout and off-switching are triggered by the same wavelength, central fluorophores are switched off during readout. As a consequence, the switching and readout sequence typically needs to be repeated in order to collect enough photons if expression levels are low. This procedure is potentially unfavorable as it increases the image acquisition time and the light dose applied to the sample.

Positive switching RSFPs can be used to overcome the problem of limited fluorescence readout per switching cycle because fluorescence excitation triggers the on-switching process and hence the proteins in the center of the doughnut can be kept in the on-state for an arbitrary time during readout. In this concept, to achieve subdiffraction resolution, the molecules in the periphery have to be kept in the off-state, which could be achieved by superposition of the regularly focused excitation light with the doughnut-shaped off-switching beam. In principle, the two overlaid beams could be scanned together over the sample to record a super-resolved image, without the requirement for sequential illumination steps. For simplicity, we refer to this approach, which is only possible with positive switching RSFPs, as one-step RESOLFT nanoscopy.

In fact, the initial demonstration of RESOLFT nanoscopy 15 years ago was performed in the one-step mode.^7^ However, the utilized protein, asFP595, showed a poor switching performance and is an obligate tetramer.^24^ It is therefore not suitable as a fusion tag in live-cell imaging applications. Consequently, subsequent realizations of the RESOLFT concept refrained from positive switching RSFPs and instead negative switching RSFPs and sequential illumination steps were used, because of the unavailability of suitable positive switching RSFPs.

Aside from asFP595,^17,24^ only a few positive switching proteins have been reported so far, and none of them display the switching performance required. rsCherry,^25^ a red-emitting RSFP, and Padron,^14^ a green-emitting RSFP engineered from Dronpa,^26^ both display a low resistance to switching fatigue and slow switching kinetics. These characteristics were improved to some extent in Kohinoor,^27^ a variant based on Padron. The recently reported Kohinoor2.0 exhibits a 2.9-fold higher molecular brightness than Kohinoor, but its switching fatigue has not been investigated and it has not been used for RESOLFT nanoscopy.^28^ Kohinoor has been used for a demonstration of point-scanning RESOLFT nanoscopy, but we found that Kohinoor is still prone to switching fatigue. However, resistance against switching fatigue is a key requirement for one-step RESOLFT imaging.

One-step RESOLFT nanoscopy requires a positive switching RSFP which does not emit fluorescence upon illumination with the off-switching wavelength. In addition, the vast majority of peripheral RSFPs need to reside in the off-state despite being irradiated with on-switching light, which requires the off-switching to dominate over the on-switching. If the equilibrium is reached quickly relative to the beam movement during sample scanning, simultaneous illumination with both superimposed beams alone should suffice to overcome the diffraction barrier.

To engineer a positive switching RSFP with the required characteristics, we chose to rely on the well-described positive switching protein Padron.^14^ This RSFP displays poor expression at 37 °C, and it features slow switching kinetics as well as high switching fatigue. On the other side, Padron displays high molecular brightness and switching contrast. Importantly, several X-ray structures of Padron are available.^18,29^ In addition, the switching mechanism of Padron has been investigated by kinetic crystallography,^18,29^ and ultrafast spectroscopy,^30,31^ at cryotemperatures^29,32^ as well as with molecular dynamics simulations,^18,33^ providing a solid database for a semirational engineering approach.

We generated the positive switching RSFP Padron2, which outperforms all previous positive switching RSFPs for RESOLFT nanoscopy. With Padron2, we established RESOLFT nanoscopy on living cells without the need for sequential switching steps.

## Results and Discussion

### Development of Padron2

All currently available positive switching RSFPs exhibit severe limitations for their application in RESOLFT nanoscopy. In order to explore possibilities of improvement, we decided to screen for improved positive switching RSFPs on the basis of Padron as a template in a semirational approach. We performed 16 rounds of mutagenesis on the Padron coding sequence. For random mutations, PCR-based error-prone mutagenesis was performed. For site-directed mutagenesis, amino acid positions were selected on the basis of previous studies investigating the effects of mutations in related proteins such as Dronpa and EosFP.^14,34,35^ Moreover, we decided to systematically mutagenize the amino acids close to the chromophore as revealed by the available structures of Padron in the on- and off-states (PDB 3LS3, 3LSA, 3ZUJ, and 3ZUL).^18,29^ After each round of mutagenesis, plasmid libraries encoding the Padron mutants were transformed into *Escherichia coli* (*E. coli*) bacteria and the bacteria were plated on agar plates. In every screening round, up to four agar plates with ∼1,000 bacterial colonies each were analyzed with an automated fluorescence microscope equipped with laser diodes for the switching of the RSFPs. This enabled repeated switching of the RSFP fluorescence with microsecond time resolution and at light intensities typically used in RESOLFT imaging. The fluorescence modulation in response to alternating illumination with light of 405 and 488 nm wavelength allowed for the determination of switching kinetics, residual off-state fluorescence intensity, switching fatigue, and effective brightness of each bacterial colony. After each screening round, the best performing variants were sequenced and chosen for further characterization and mutagenesis.

Finally, we identified a Padron variant with substantially improved properties that differed at 11 positions from its template Padron: M40V, T58S, R66K, A69I, S82L, Y114F, L141P, F173S, S190A, E218G, and R221G (Supporting Information Figures S1 and S2). Several, but not all of these positions had been identified in previous studies aiming at modifying fluorescent proteins. The positions R66 and A69 were shown to affect the protonation state of the chromophore and the transition to dark states in photoconvertible FPs.^36^ The mutation L141P was demonstrated to shift the protonation state of the chromophore.^18^ A study on the multiphotochromic FP IrisFP demonstrated that the mutation F173S results in a less dense chromophore pocket, which facilitates cis–trans isomerization.^37^ Because previously it had been reported that N- and C-termini matching those of EGFP improve the tagging performance of FPs,^38^ we also engineered the N- and C-termini accordingly. We named this engineered RSFP Padron2.

### General Properties of Padron2

**Figure 1:**
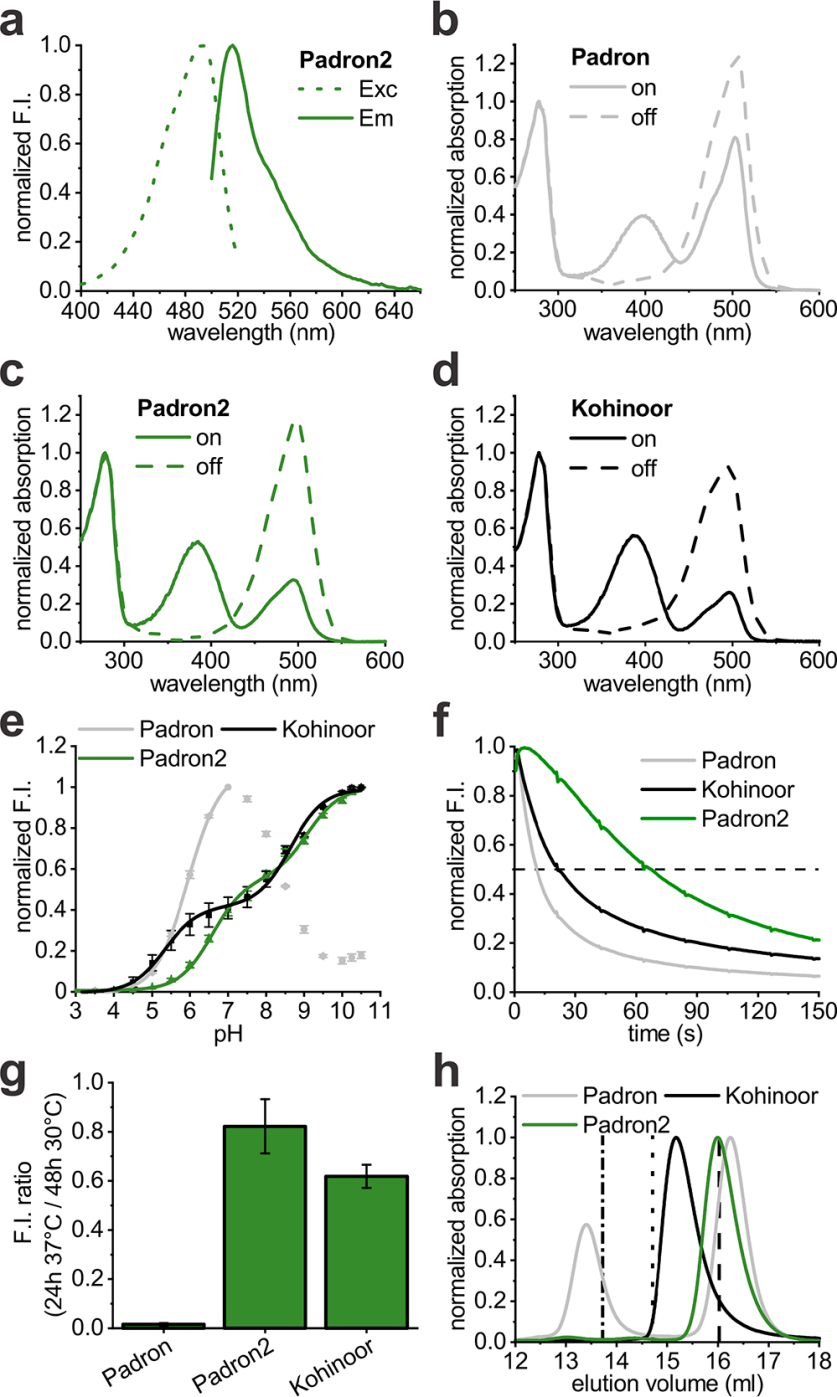
Protein characteristics. (a) Excitation (Exc) and emission (Em) spectra of Padron2. (b–d) Absorption spectra of Padron (b), Padron2 (c), and Kohinoor (d) in the ensemble on- and off-states. (e) pH-dependent normalized fluorescence intensity of purified protein solutions at the equilibrated state. (f) Photobleaching in bacterial colonies under 488 nm illumination. (g) Fluorescence intensity ratios measured in bacterial colonies after 24 h growth at 37 °C and 48 h growth at 30 °C. For absolute fluorescence intensity values, see Supporting Information Figure S3d. (h) Size exclusion chromatography of purified protein samples. Absorption was measured at 280 nm; vertical lines indicate the peak elution volume of tetrameric DsRed (dashed–dotted line), dimeric dTomato (dotted line), and monomeric mEGFP (dashed line). F.I., fluorescence intensity.

To evaluate the properties of Padron2, we systematically compared it to Padron and Kohinoor. Padron2 has a fluorescence excitation maximum at 492 nm and an emission maximum at 516 nm (Figure 1a, Table 1). Thus, it is spectrally slightly blue-shifted in comparison to Padron and it is similar to Kohinoor in this respect. The fluorescence lifetime of Padron2 is slightly shorter compared to Padron and Kohinoor (3.0 ns *vs* 3.4 and 3.5 ns) (Table 1). Kept in the dark at pH 7.5, ∼67% of Padron2 molecules adopted the on-state, while Padron and Kohinoor predominantly resided in the off- or on-state, respectively (Table 1; Supporting Information Figure S3a–c).

**Table 1:** Protein Characteristics of Padron, Padron2, and Kohinoor

	exptl conditions	Padron	Padron2	Kohinoor
absorption max (nm)	on-state (neutral, anionic)	398, 504	384, 495	388, 496
	off-state	505	498	496
excitation max (nm)^a^	equilibrated, pH 7.5	502	492	496
emission max (nm)^a^	equilibrated, pH 7.5	522	516	518
emission max (nm)	on-state, pH 7.5	519	513	514
extinction coeff at abs max (M^–1^ cm^–1^)				
anionic on-state	pH 7.5, on-state	32,700 ± 500^b^	13,250 ± 700	12,400 ± 600^c^
neutral on-state	pH 7.5, on-state	19,200 ± 900	23,500 ± 1,000	24,800 ± 1,500
off-state	pH 7.5, off-state	57,700 ± 2,100	48,700 ± 4,800	39,500 ± 1,000
fraction of anionic on-state chromophore (%)	in solution, pH 7.5	58.2	22.2	18.0
on-switching quantum yield	in solution, pH 7.5	na	0.005 ± 0.002	0.015 ± 0.002^d^
off-switching quantum yield	in solution, pH 7.5	na	0.115 ± 0.003	0.088 ± 0.011^e^
quantum yield	on-state, pH 7.5	0.64^f^	0.49 ± 0.02	0.73 ± 0.03^g^
molecular brightness	on-state, pH 7.5	20.9	6.5	9.1
p*K*_a_	equilibrated, in solution	5.9	6.6, 9.1	5.3^h^, 8.6
time to bleach to 50% F. I. (s)	in bacterial colonies	11.4	67.4	21.5
fluorescence lifetime (ns)	in solution, pH 7.5	3.4 ± 0.02	3.0 ± 0.05	3.5 ± 0.09
equilibrium F. I. (% of on-state)	in solution, pH 7.5	8.9 ± 1.2	66.7 ± 2.4	96.7 ± 1.8
residual fluorescence intensity in the ensemble off-state (% of on-state)	in solution, pH 7.5	4.0 ± 0.3	1.9 ± 0.1	2.0 ± 0.1
residual fluorescence intensity in the ensemble off-state (% of on-state)	in bacterial colonies	2.27 ± 0.02	1.41 ± 0.09	8.42 ± 0.63
switching half-time (ms)^i^	in bacterial colonies	81.9	32.8	37.6
no. of cycles at 50% maximal intensity	in bacterial colonies	19 (113^j^)	990 (321^k^)	37 (182^j^)
maturation half-time (min)	purified solution	na	∼70	∼160^l^

a For spectra, see Figure 1a and Supporting Information Figure S3e,f.

b Published: 43,000.^14^

c Published: 62,900 ± 136, measured at pH 10.^27^

d Published: 0.02.^27^

e Published: 0.15.^27^

f Published value.^14^

g Published: 0.71 ± 0.05.^27^

h Published: 5.9 and 8.6.^27^

i Measured at 1.3 kW/cm^2^ 488 nm intensity.

j Measured with Padron2 settings.

k Measured with Kohinoor settings.

l Published value;^27^ F. I., fluorescence intensity.

Switched to the on-state, Padron2 molecules in solution displayed two distinct absorption bands at 384 and 495 nm (Table 1), resulting from the protonated and deprotonated forms of the chromophore.^10,15^ Irradiation of the on-state Padron2 with 405 nm transfers the proteins into the off-state, while irradiation with light of 488 nm induces fluorescence.

In on-state Padron2 and on-state Kohinoor, the equilibrium between the neutral, protonated chromophore and the anionic, deprotonated chromophore was shifted toward the protonated chromophore compared to Padron (Figure 1b–d; Table 1).

Concretely, in the on-state equilibrium at pH 7.5, about 60% of the Padron chromophores are in the emissive (deprotonated) state, but only about 20% of the chromophores of Padron2 and Kohinoor are deprotonated (Table 1). The lowered occupancy of the deprotonated on-state contributed to the decrease in molecular brightness (*i.e.*, the product of extinction coefficient and quantum yield divided by 1,000) of on-state Padron2 (6.5) and Kohinoor (9.1) at pH 7.5 compared to Padron (20.9) (Table 1), but also resulted in a higher off-switching rate. For Kohinoor a molecular brightness of 44.7 has previously been reported.^27^ However, this value was determined at an unphysiological pH value of 10.0 and is hence irrelevant for an adequate comparison. Padron has a p*K*_a_ of 5.9 (Table 1) and fluorescence decreases strongly at pH values above 7.0 (Figure 1e). Since pH values between 7 and 8 are common in cells, this is an undesirable property. This disadvantage is eliminated in Padron2, which is stable at pH values as high as 11.0 (Figure 1e; Supporting Information Figure S4), with two p*K*_a_ values of 6.6 and 9.1 (Table 1). Padron and Kohinoor have lower p*K*_a_ values than Padron2; consequently, Padron2 is less bright at acidic conditions. The two p*K*_a_ values of Padron2 and Kohinoor and the pH-dependent changes in the absorption spectra (Figure 1e; Supporting Information Figure S4) indicate more than one protonation site.

We also determined the quantum yields for on- and off-switching of Padron2. The on-switching quantum yield of Padron2 (0.005) is three times lower than that of Kohinoor (0.015), whereas the off-switching quantum yield is slightly higher (0.115 *vs* 0.088).

An important parameter for the usability of a fluorescent protein in microscopy is its stability against photobleaching. Under continuous illumination at 488 nm with 2.3 kW/cm^2^ in bacterial colonies, Padron2 displayed a 3- and 6-fold increased resistance to photobleaching in comparison to Kohinoor and Padron (Figure 1f, Table 1), respectively.

Padron exhibits poor expression at 37 °C but is well-expressed at lower temperatures. To evaluate the expression of Padron2 at 37 °C, we compared the fluorescence of bacterial colonies grown for 24 h at 37 °C with colonies grown for 48 h at 30 °C (Figure 1g; Supporting Information Figure S3d). Whereas *E. coli* colonies expressing Padron2 showed almost the same fluorescence signal under both conditions, Kohinoor exhibited a significantly weaker fluorescence signal upon growth at 37 °C and Padron expressing colonies exhibited almost no mature protein at 37 °C. In line with this observation, at 37 °C, the maturation half-time of Padron2 was ∼70 min (Table 1, Supporting Information Figure S5), whereas for Kohinoor a maturation half-time of ∼160 min was reported.^27^ We could not determine the maturation half-time of Padron, because of its poor expression at 37 °C.

Taken together, with regard to key spectroscopic properties used to characterize fluorescent proteins *in vitro*, Padron2 is similar to Padron and Kohinoor, and with respect to photostability and bacterial expression at 37 °C, it outperforms these RSFPs.

### Tagging with Padron2

In size exclusion chromatography, Padron2 eluted as a monomer (Figure 1h), suggesting its usability as a fusion tag. In comparison, Padron eluted in two distinct populations as tetramer as well as a monomer, suggesting slow dissociation kinetics (Figure 1h).^39^ Kohinoor eluted between the volumes of a dimer and a monomer, indicating fast dissociation and association rates of a dimeric interaction under these conditions.^39^

**Figure 2:**
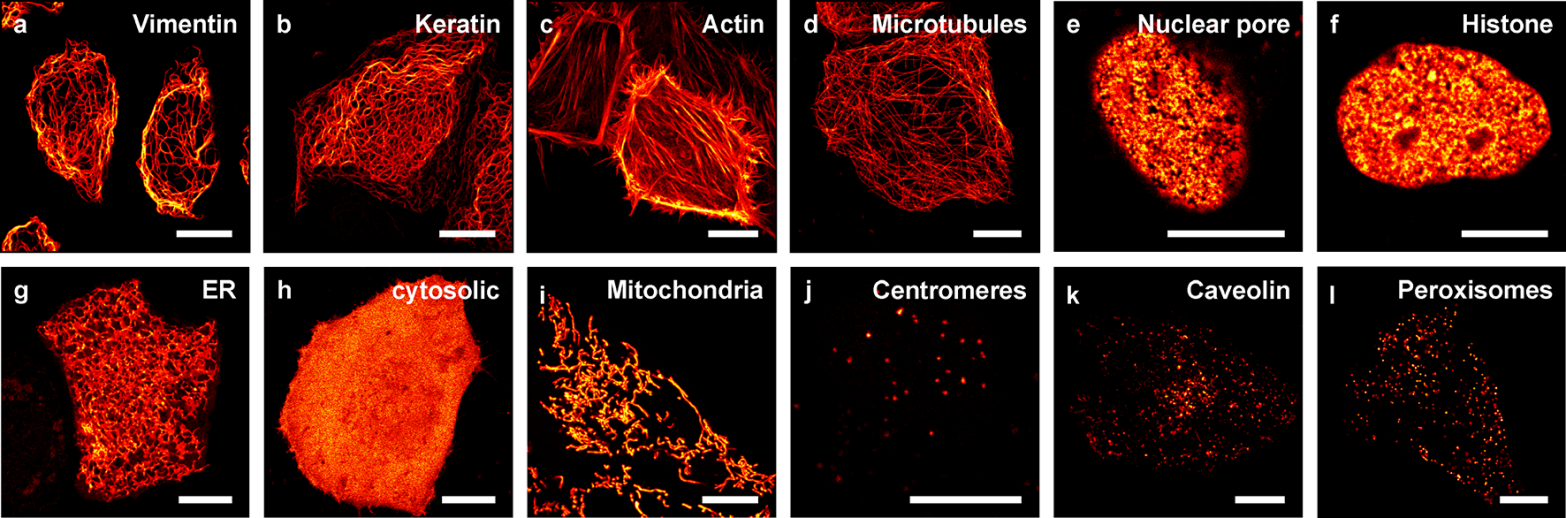
Padron2 fusion constructs in HeLa cells. The fusion proteins were transiently expressed in HeLa cells. (a) Vimentin (vimentin-Padron2), (b) keratin (keratin18-Padron2), (c) actin (lifeact-Padron2), (d) microtubules (Map2-Padron2), (e) nuclear pore (Padron2-Nup50), (f) histone (Padron2-histone H2bn), (g) endoplasmic reticulum (TS-Padron2-ER retention signal) (h) cytosolic, (i) mitochondria (TS-Padron2), (j) centromeres (Padron2-CenpC), (k) caveolin (caveolin-1-Padron2), and (l) peroxisomes (Padron2-TS). Images were recorded 24 h post transfection in a single plane (e–h, j–l) or are shown as maximal projection of a *z*-stack (a–d, i). TS, targeting sequence. Scale bars: 10 μm.

To analyze the performance of Padron2 as a tag in fusion proteins, we cloned several Padron2 fusion constructs targeted to various cellular structures and expressed them in human HeLa cells (Figure 2). All Padron2 fusion constructs tested localized correctly. Constructs included fusions to vimentin (VIM), keratin 18 (KRT18), the actin binding peptide lifeact, the microtubule associated protein Map2 (MAP2), the centromere protein C1 (CENPC1), caveolin 1 (CAV1), and the nuclear pore complex protein nucleoporin 50 (NUP50) as well as to the histone H2bn (HIST1H2BN). We also targeted Padron2 successfully to the lumen of the ER, the mitochondrial matrix, peroxisomes, and the cytosol (Figure 2).

### Switching Characteristics of Padron2

In RESOLFT imaging, three switching parameters of an RSFP are of key relevance: (1) switching kinetics, (2) residual fluorescence intensity in the ensemble off-state, and (3) switching fatigue, which is the switching induced photodestruction of the fluorophore. The switching fatigue is influenced by the actual photobleaching, but also by other factors including the switching speed, which directly relates to the required light dose for on- and off-switching per cycle. Padron and Kohinoor are particularly poor with respect to switching fatigue, so we primarily screened for a variant that showed a substantially higher resistance against switching fatigue, while at the same time we aimed at generating an RSFP that enabled a faster off-switching and a lower residual fluorescence background than Padron.

**Figure 3:**
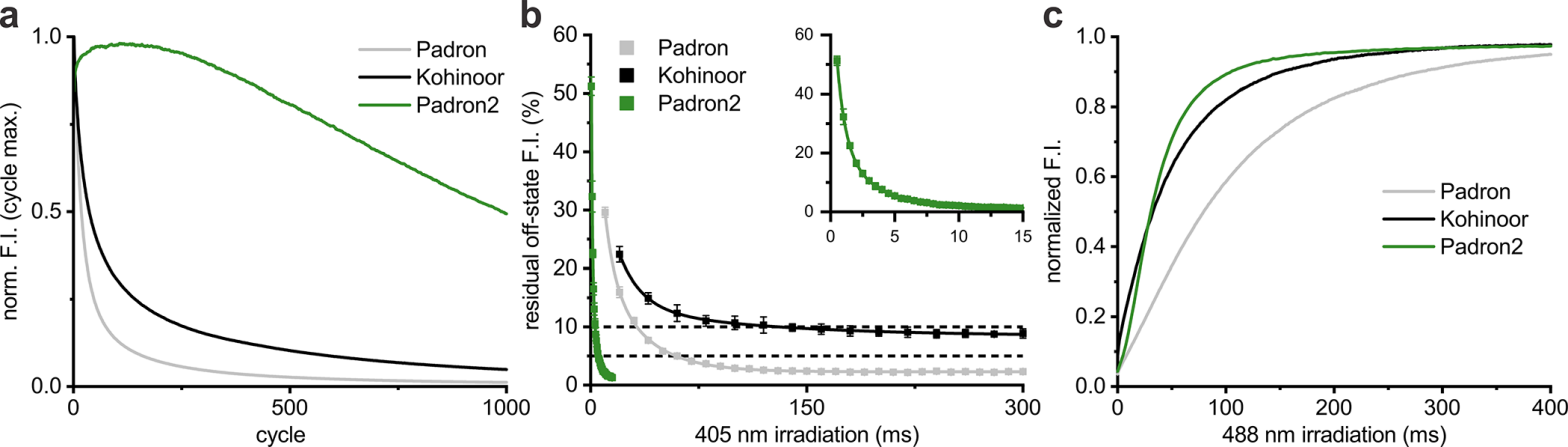
Switching performance. (a) Switching fatigue in bacterial colonies. Line graphs represent maximal fluorescence intensities of the on-switching curves of every cycle. (b) Residual fluorescence intensity in bacterial colonies after different 405 nm illumination times with an intensity of 4.1 kW/cm^2^. Squares represent averaged measurements, while the line graph is the respective exponential decay fit. Inset graph displays Padron2 data from the main graph with adjusted axis scales. (c) Normalized on-switching curves in bacterial colonies. Padron and Padron2 had previously been switched to 5% residual fluorescence intensity in the ensemble off-state, while Kohinoor had been switched to 10%. F. I., fluorescence intensity.

To compare the switching fatigue of Padron2, Padron, and Kohinoor, we cycled, using sequential illumination, the fluorescence of the respective proteins expressed in *E. coli* between 5% residual fluorescence intensity (10% in the case of Kohinoor because it could not be switched below 8%) and 95% of the full on-state by choosing appropriate light intensities and illumination times. The fluorescence of bacterial colonies expressing Padron2 was cycled almost 1,000 times before the fluorescence intensity was bleached to 50%. With Padron and Kohinoor, we achieved only 19 and 37 cycles, respectively (Figure 3a; Supporting Information Figure S6; Table 1). Hence, Padron2 displayed an outstanding resistance against switching fatigue upon sequential illumination with light of 405 and 488 nm.

One-step RESOLFT microscopy entirely refrains from sequential illumination steps, as a regularly focused excitation beam of 488 nm is superimposed with a doughnut-shaped beam of 405 nm light. In this concept, RSFPs in the periphery of the excitation focus are predominantly present in the off-state, because off-switching dominates over the on-switching process. Toward the central focal region, where the 405 nm light intensity is at a minimum, the intensity of light of 488 nm increases. Hence, toward the center of the doughnut of 405 nm light, increasingly more RSFPs reside in the on-state and emit fluorescence. By tuning the relative light intensities of 405 and 488 nm, the size of the central, fluorescent region can be adjusted to a subdiffraction sized volume. This approach to RESOLFT nanoscopy requires a positive switching RSFP, which is mostly (≥95%) present in the off-state, when simultaneously irradiated with light of 405 and 488 nm of appropriate intensities. Presumably, when irradiated with both wavelengths, the positive switching RSFP will cycle between the on- and the off-states. Hence, a suitable RSFP is required to display a good resistance against switching fatigue upon simultaneous irradiation with two wavelengths. To investigate the behavior of Padron2 at this condition, we irradiated bacterial colonies expressing Padron2, Padron, or Kohinoor with light of both 405 and 488 nm simultaneously. Intensities were chosen so that the off-switching process was dominant and the proteins were effectively switched off, a situation that would be required for one-step RESOLFT nanoscopy. In this experiment, Padron2 displayed faster off-switching rates as well as lower residual fluorescence intensities than both Padron and Kohinoor (Supporting Information Figure 7). In addition, photodestruction of Padron2 was very low in comparison to Padron and Kohinoor, when illuminated with both wavelengths at the same time (Supporting Information Figure 8). Together these observations suggested that Padron2, but neither Padron nor Kohinoor, would be suitable for RESOLFT nanoscopy without sequential illumination steps.

Next, we aimed at quantitatively comparing the off-switching speed of Padron2, Padron, and Kohinoor using the same light intensities. Padron2 shows very little fluorescence when being excited by light of 405 nm, the wavelength that induces the transition from the on- to the off-state. This property renders it challenging to probe the off-switching speed, as one cannot measure the decrease of fluorescence upon illumination with 405 nm light. To overcome this challenge, bacterial colonies expressing the RSFP were first irradiated with light of 405 nm for varying time periods (between 0 and 300 ms), and subsequently the on-switching by light of 488 nm was recorded. From these responses, we determined the relative residual fluorescence intensity to which the fluorophores were transferred (Figure 3b). At an intensity of 4.1 kW/cm^2^ of 405 nm light, Padron2 was switched to a residual fluorescence intensity of 5% within 5.3 ms, while switching of Padron required nearly 12 times longer illumination to be switched to the same value. The fluorescence of bacterial colonies expressing Kohinoor could not be switched to a residual fluorescence intensity below 8.2%, and switching times to achieve this value were 2 orders of magnitude longer than those required for Padron2.

The ensemble off-switching speed of Padron2 increased with higher 405 nm intensity (Supporting Information Figure S9a). This is an important beneficial feature of RSFPs used for RESOLFT nanoscopy, as a predictable response of the switching kinetics to increasing light intensities enhances the robustness of the imaging protocol and allows one to shorten the imaging dwell time by increasing the laser intensity, if required. Increasing the 405 nm light intensity enabled us to reach residual fluorescence intensities in the ensemble off-state below 1% with Padron2 (Supporting Information Figure S9a). In contrast, increasing the 405 nm laser intensity was not practical when using Padron or Kohinoor, as it resulted in increased residual fluorescence intensities (Supporting Information Figure S9b,c).

With respect to on-switching, Padron2 exhibits a fast switching half-time of 32.8 ms at 488 nm (1.3 kW/cm^2^). Thereby it is slightly faster than Kohinoor (37.6 ms) and strongly outperforms Padron (81.9 ms) (Figure 3c; Table 1).

Taken together, Padron2 outperforms both Padron and Kohinoor at all tested switching conditions. Most notably, it exhibits an outstanding resistance against switching fatigue as well as fast and robust switching to the off-state.

### RESOLFT Nanoscopy

**Figure 4:**
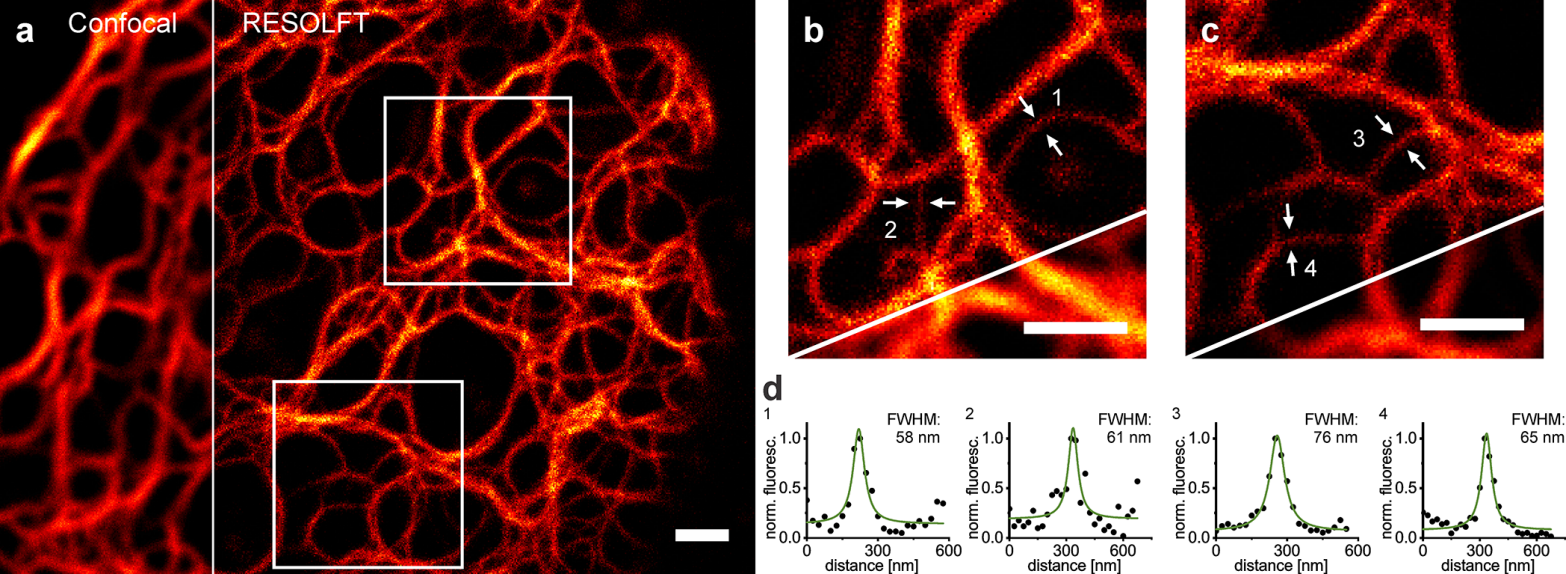
Confocal microscopy and RESOLFT nanoscopy image of vimentin-Padron2 fusion constructs. (a) Confocal and RESOLFT overview image. (b, c) Magnifications of panel a. (d) Intensity line profiles of the positions indicated by arrows. HeLa cells were transiently transfected with the expression plasmid and were imaged at ambient temperature 24 h post-transfection. The RESOLFT image was recorded with sequential illumination with 70 μs of 488 nm activation with a regularly focused beam, 350 μs off-switching with a doughnut-shaped 405 nm beam, and 120 μs of 488 nm readout with a regularly focused beam. Pixel size: 25 nm. Images show raw data. Line profiles were measured across three adjacent pixels and were fitted with a Lorentzian function. Scale bars: 1 μm.

To test if Padron2 is a suitable probe for RESOLFT imaging, we first expressed Padron2 fused to the intermediate filament protein vimentin (VIM-Padron2) and applied a sequential illumination RESOLFT imaging scheme. We were able to perform RESOLFT nanoscopy on living HeLa cells (Figure 4). On the vimentin filaments we measured a full width at half-maximum (FWHM) of around 60 nm. This value is slightly higher than previously demonstrated on similar samples expressing the negative switching RSFP rsEGFP2 fused to vimentin and similar to the reported FWHMs measured with rsGreenF and rsFolder2.^11,40,41^ Clearly, RESOLFT nanoscopy with Padron2 enabled a resolution better than the diffraction limit (∼180 nm).

**Figure 5:**
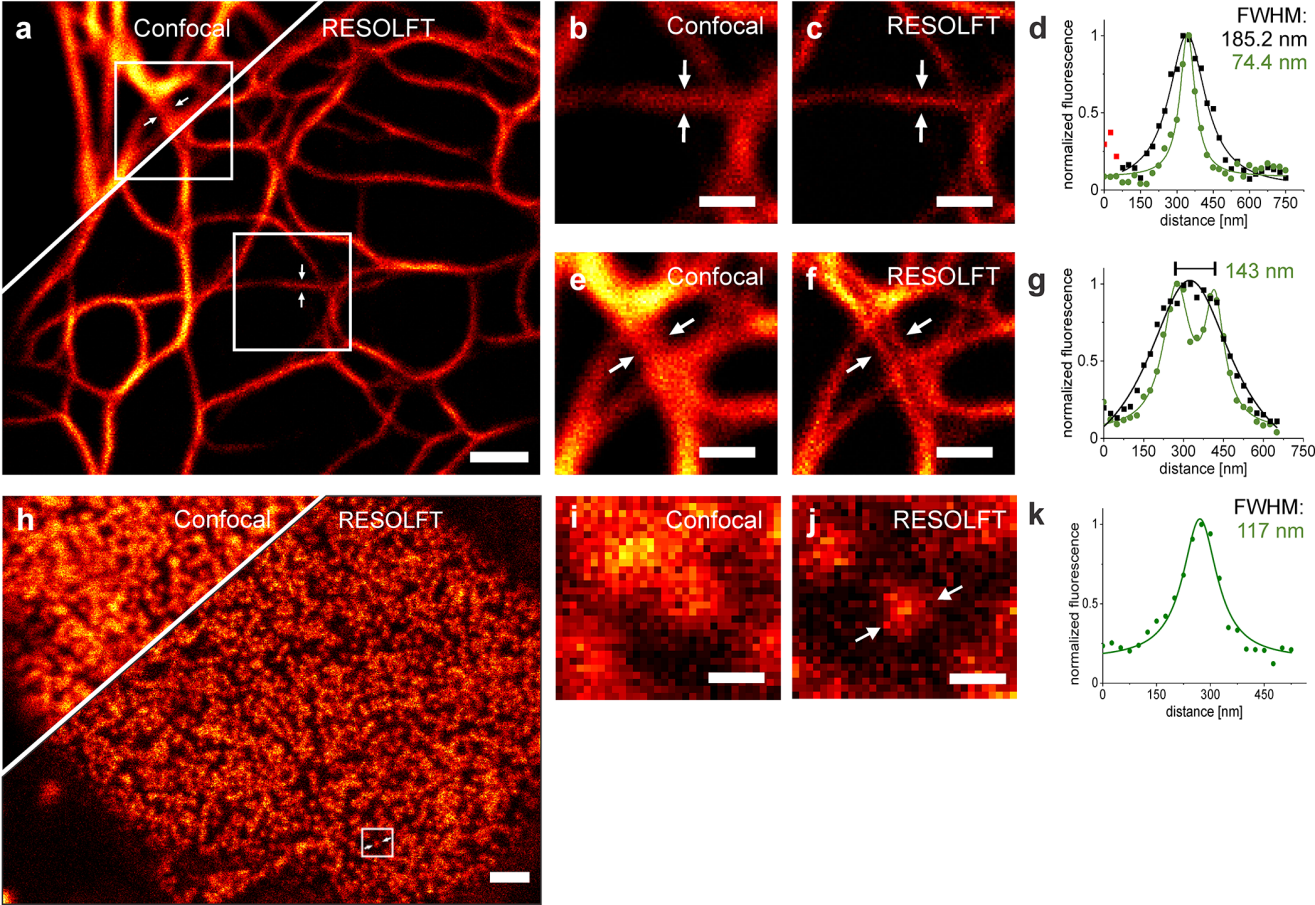
Confocal microscopy and one-step RESOLFT nanoscopy images of Padron2 fusion constructs. (a) Confocal and RESOLFT overview image of vimentin-Padron2. (b, c, e, f) Magnifications of the areas indicated in panel a. (d, g) Intensity line profiles across three adjacent pixels determined at the indicated sites. Data points were fitted with a Lorentzian function. Black, confocal; green, RESOLFT. (h) Confocal and RESOLFT overview image of Padron2-Nup50. (i, j) Magnifications of the areas indicated in panel h. (k) Intensity line profile measured across three adjacent pixels at the indicated sites. HeLa cells were transiently transfected with the respective expression plasmid and imaged at ambient temperature 24 h post-transfection. The RESOLFT image in panel a was recorded with a pixel dwell time of 300 μs with constant superimposed illumination of a regularly focused 488 nm laser at 1.4 kW/cm^2^ and a doughnut-shaped 405 nm laser at 1.0 kW/cm^2^. The RESOLFT image in panel h was recorded with a pixel dwell time of 500 μs with constant superimposed illumination of a regularly focused 488 nm laser at 1.7 kW/cm^2^ and a doughnut-shaped 405 nm laser at 0.9 kW/cm^2^. Pixel size: 25 nm. All images show raw data. Scale bars: 1 μm (a, h), 0.5 μm (b, c, e, f,), and 0.2 μm (i, j).

Next, we performed RESOLFT nanoscopy without sequential illumination steps on the VIM-Padron2 expressing cells and the resulting images also revealed details that were concealed in the corresponding diffraction-limited confocal images (Figure 5a). Here, we typically measured a FWHM of ∼75 nm (Figure 5b–d) and we were able to resolve adjacent filaments that were as close as ∼140 nm (Figure 5e–g).

We additionally fused Padron2 to the nuclear protein Nup50, which is located within the basket of the nuclear pore (Padron2-NUP50).^42^ Padron2-Nup50 expressed at lower levels than VIM-Padron2. To be able to image these comparatively dim samples, we made use of the fact that in the one-step RESOLFT mode the pixel dwell-time can be prolonged to collect more photons, if required. We utilized this property and prolonged the pixel dwell time from 300 to 500 μs (Figure 5h–k). This allowed us to record Padron2-Nup50 in cells, demonstrating that this simplified RESOLFT approach can be used to visualize less abundant proteins at subdiffraction resolution in living cells.

### PSF Calculations

In order to determine, if the experimentally determined resolutions correspond to the theoretically attainable resolutions, we calculated the point-spread functions (PSFs) and the resulting predicted FWHM when imaging Padron2. To this end, we used the experimentally determined photophysical properties of Padron2, specifically the quantum yields for switching (Table 1), as well as the extinction coefficients at 488 and 405 nm (Supporting Information Table S1), as these are the wavelengths used in the RESOLFT microscope. For the model, we assumed the light intensities that were experimentally identified as most suitable. Although in the calculations, higher light intensities would result in a predicted higher resolution, in the experiments higher light intensities had undesirable effects such as increased photobleaching. The calculations suggest that using the experimental light intensities and imaging conditions an FWHM of the effective PSF of 54 nm in the case of the sequential imaging scheme and of 60 nm in the case of one-step RESOLFT can be expected. Given the fact that a vimentin filament has a physical extent and that the calculations were performed for ideal conditions assuming the absence of any noise, these calculated data are in good accordance with the measured FWHM values on vimentin filaments (60 and 75 nm, respectively). The calculations demonstrate that in the case of the sequential illumination scheme the effective PSF exhibits a stronger decline and a lower intensity at the periphery (Supporting Information Figure S10), which is expected to result in less background signal.

## Conclusions

We have shown in this work that Padron2 is usable for RESOLFT nanoscopy. Previously, negative switching RSFPs, such as rsEGFP2,^11^ have outperformed the available positive switching RSFPs with respect to most relevant characteristics. Padron2 exhibits several properties that are close to those of negative switching RSFPs. It is highly resistant against switching fatigue as it sustains half of the switching cycles reported for rsEGFP2. The ensemble switching speeds of both RSFPs are comparable, and with Padron2 a much lower ensemble residual off-state fluorescence intensity could be reached than the one reported for rsEGFP2.

These improvements were at the cost of molecular brightness, as this has been reduced in Padron2 by a factor of 3.2 or 1.4 compared to Padron or Kohinoor, respectively. However, this is outweighed by its improved expression at 37 °C, a key requirement for the use of a genetically encoded probe in mammalian cells.

The on-state of Padron2 consists of an equilibrium of protonated and deprotonated chromophores, with absorbance bands at 384 and 495 nm, which enables efficient off-switching. Due to this equilibrium, which is prevalent in all positive switching RSFPs, the molecular brightness of positive switching RSFPs is in many cases lower than that of negative switching RSFPs, as in these proteins the on-state is typically fully deprotonated. This on-state protonation equilibrium results in a higher p*K*_a_ than is found in most negative switching RSPSs, which favors imaging with Padron2 at neutral or alkaline conditions; the high p*K*_a_ may represent a challenge when Padron2 is in an acidic environment.

In this work, we showed that Padron2 can be efficiently switched into the off-state while illuminated simultaneously with light of 405 and 488 nm. We demonstrated that this fact can be exploited to implement RESOLFT nanoscopy without sequential switching steps by just scanning the sample using an excitation beam overlaid with a doughnut-shaped off-switching beam. This reduces the technical complexity of the RESOLFT setup, and presumably of more relevance, it reduces the number of parameters that need to be optimized for recording a RESOLFT image. We also observed that the simultaneous irradiation reduced the photobleaching compared to sequential irradiation, when comparable image quality was aimed at (Supporting Information Figure S11).

We speculate that in particular parallelized RESOLFT imaging or variants thereof^19,43,44^ will benefit from the properties of Padron2, as in this approach the actual dwell time has less influence on the overall recording time and switching steps generally complicate the imaging. Such approaches are likely to benefit from the fact that with Padron2 the dwell time can be prolonged without the RSFP being switched off by the excitation light. In addition, Padron2 might be useful for imaging schemes that exploit the coupling of two switching mechanisms such as protected STED.^45^

A fundamental challenge to all live-cell imaging, and in particular to super-resolution microscopy, is to prevent phototoxic light intensities and doses.^12,13^ For RESOLFT microscopy with green fluorescent proteins, light of 405 and 488 nm is used. In the one-step RESOLFT approach utilized in this study, the 488 nm light dose applied was about ten times lower than typically used in sequential mode RESOLFT microscopy with rsEGFP2.^11,46^ On the contrary, the 405 nm light dose was about five times higher than typically used in sequential mode RESOLFT microscopy with negative switching RSFPs. We were able to record consecutive images of living cells. Still, the higher 405 nm light dose is potentially harmful, and it will require further investigations to quantify potential phototoxic effects. Such effects could be addressed by using imaging schemes that adapt the scanning and the applied light doses to the actual sample.^47^ In fact, positive switching RSFPs are particularly suited for approaches to reduce the 405 nm light dose, as the 405 nm illumination is not required for the on-switching process.

Currently, the optical resolution and the image quality obtained with Padron2 is not as good as typically achieved with rsEGFP2 in sequential RESOLFT. We expect that further developments on Padron2 and parallelized imaging schemes dedicated to explore the benefits of positive switching RSFPs will change this situation.

Altogether, we engineered in this study the positive switching RSFP Padron2 and achieved improvements with regard to properties that were identified as rather unfavorable in established green fluorescent positive switching RSFPs, while maintaining the beneficial properties of its template Padron. Padron2 specifically exhibits strong resistance against switching fatigue, low photobleaching in the on-state and fast ensemble switching. We demonstrated its use for live-cell RESOLFT nanoscopy.

## Methods

### Mutagenesis

Coding sequences were mutagenized by site-directed-saturation (QuickChange protocol, Stratagene, La Jolla, Ca, USA), error-prone,^48^ or multiple-site mutagenesis.^49^ GFPends were introduced *via* PCR and reinsertion of the coding sequence into the backbone *via* restriction with enzymes *Eco*RI and XhoI (forward primer, ACGGATCCAATGGTGAGCAAGGGCGAGGAGAACAACATGGCCGTGATTAAACCAGAC; reverse primer, ATTAAGCTTCGAATTCTTACTTGTACACTCGTCCATGGCCTGCCTCGGCAG).

### Screening

Mutant libraries of early variants were generated in the pQE-31 expression vector (Qiagen, Hilden, Germany) and expressed in SURE *E. coli* (Stratagene) on LB agar. Mutant libraries of later variants were generated in the pBAD/HisB expression vector from pBAD-mKalama1, which was a gift from Robert Campbell (Addgene plasmid no. 14892).^50^ Libraries were transformed into One Shot TOP10 Electrocomp *E. coli* (Thermo Fisher Scientific, Waltham, MA, USA) and plated on LB agar with 0.02% (w/v) arabinose. Bacterial colonies were screened with a customized DM5500B microscope (Leica Microsystems, Wetzlar, Germany) with a SCAN 100 × 100 stage (Märzhäuser Wetzlar, Wetzlar, Germany). Laser sequences were controlled with an FPGA program based on LabVIEW (National Instruments, Austin, TX, USA).

### Protein Purification

Padron, Padron2, and Kohinoor were expressed in One Shot TOP10 Electrocomp *E. coli* (Thermo Fisher) from the pBAD/HisB expression system. To this end, the coding sequence for Kohinoor was PCR amplified from Kohinoor/pRSETb (forward primer, AGGGCTCGAGCATGAGTGTGATTAAACC; reverse primer, AACGAATTCTTACTTGGCCTGCCT), which was a gift from Takeharu Nagai (Addgene plasmid no. 67770), and inserted into pBAD/HisB with restriction enzymes *Eco*RI and XhoI. After 24 h at 37 °C (Padron2, Kohinoor) or 48 h at 30 °C (Padron) growth on LB agar with 0.02% (w/v) arabinose, cultures were kept at room temperature overnight to ensure full maturation. Cells were collected in 20 mM phosphate, 500 mM NaCl, and 20 mM imidazole (pH 7.4). Cell suspensions were incubated on ice for 4 h with 1 mg/mL lysozyme (Serva electrophoresis, Heidelberg, Germany). After incubation, complete protease inhibitor (Roche, Basel, Switzerland) was added and samples were frozen and thawed 5× in liquid nitrogen and lukewarm water. Lysates were then centrifuged with 0.5 μL of benzonase (Thermo Fisher) added per 2 mL at 4 °C and 21,000 rcf for 3–6 h. RSFPs were isolated from the supernatant with the His SpinTrap kit (GE Healthcare, Chicago, IL, USA) and concentrated with Vivaspin 500 centrifugal concentrators with a molecular weight cutoff of 10,000 kDa (Sartorius, Göttingen, Germany). Elution buffer was exchanged to standard Tris protein buffer (100 mM Tris and 150 mM NaCl at pH 7.5) with NAP-5 columns (GE Healthcare), and samples were concentrated as before.

### Size Exclusion Chromatography

Protein samples were diluted to 10 μM and equilibrated at 6 °C overnight. A 250 μL aliquot was applied to an Äkta pure chromatography system equipped with a Superdex 200 Increase 10/300 GL column (GE Healthcare) and run at 6 °C. The flow rate was 0.75 mL/min, and protein elution was monitored with a U9-L UV monitor (GE Healthcare) at 280 nm.

### Spectroscopy

Purified protein samples were diluted to an absorption of 0.1 at 280 nm, corresponding to a concentration of approximately 20 μM. Samples were equilibrated overnight at 21 °C. Absorption spectra were recorded with a Cary 4000 UV–VIS spectrophotometer (Varian, Palo Alto, CA, USA) in an ultra-micro fluorescence cell cuvette with a 1.5 mm light path (Hellma, Müllheim, Germany). Spectra were normalized to absorption at 280 nm. Emission spectra of switching states (Supporting Information Figure S3a–c) were measured in the same cuvette with a Cary Eclipse fluorescence spectrophotometer (Varian) with excitation at 460 nm. Switching to the on- or the off-state was facilitated in a cuvette with a mercury-vapor lamp with a HQ405/10 X filter for off-switching (9.9 mW/cm^2^, 0.74 mW measured behind the cuvette filled with Tris protein buffer) and a ET500/20 X filter for on-switching (18.7 mW/cm^2^, 1.4 mW measured behind the cuvette filled with Tris protein buffer). Proteins were switched for 3–5 min until saturation. Power was measured with a PM200 power meter equipped with a S170C sensor (ThorLabs, Newton, NJ, USA). The spectra recorded this way were integrated and used for the determination of the residual fluorescence intensity in the ensemble off-state and the equilibrium state fluorescence intensity in solution.

The extinction coefficients of the on- and off-states of RSFPs were determined using the alkaline denaturation method.^51,52^ Purified RSFPs were diluted to an 80 μM stock solution in 100 mM Tris pH 7.5 buffer, equilibrated overnight at 21 °C, and switched into the respective on- and off-states (on-switching, ET500/20 × 12.61 mW/cm^2^; off-switching, HQ405/10 X filter, 6.02 mW/cm^2^). An absorbance spectrum of the intact protein was recorded after the switched protein solution was mixed with an equal amount of Tris protein buffer pH 7.5 and transferred immediately to the cuvette. An absorbance spectrum of the denatured proteins was recorded after the switched protein solution was mixed with an equal amount of 2 M NaOH and transferred immediately to the cuvette. The extinction coefficients of the intact RSFPs were calculated by assuming that the peak extinction coefficient value of the denatured protein was equal to the extinction coefficient of the NaOH-denatured GFP chromophore, *i.e.*, 44.000 M^–1^ cm^–1^ at 447 nm.

Quantum yield was calculated from the integrated on-state emission spectra relative to the quantum yield of Padron following eq 1.1. Three to four spectra were used for the calculation, and results were averaged.
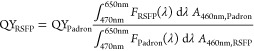
1.1(QY, quantum yield; *F*(λ), fluorescence emitted with the wavelength λ; *A*, absorption).

The excitation and emission spectra in Figure 1a and Supporting Information Figure S3e,f and pH spectra were recorded in 96 well UV-Star microplates (Greiner Bio-One, Frickenhausen, Germany) with the Cytation 3 imaging plate reader (BioTek, Winooski, VT, USA). Samples were diluted to 200 μM, equilibrated at 21 °C overnight, and diluted to 200 μL of 5 μM in triplicate wells prior to measurements. Several buffers in 0.5 pH steps were used for dilution (pH 3.0–5.5:100 mM citric acid, 150 mM NaCl; pH 6.0–7.0: 100 mM KH_2_PO_4_, 150 mM NaCl; pH 7.5–8.5: 100 mM Tris, 150 mM NaCl; pH 9.0–10.5 (+10.25): 100 mM glycine, 150 mM NaCl). Emission spectra were recorded with 470 nm excitation from 500 to 700 nm and excitation spectra from 400 to 520 nm with emission detection at 550 nm. Detector sensitivity was scaled to the brightest well. For the p*K*_a_ calculation, fluorescence was recorded with at 485/20 excitation filter and a 528/20 emission filter. Fluorescent pH response was fitted to a mono- (Padron; eq 1.2) or biphasic (Padron2, Kohinoor; eq 1.3) dose response function.monophasic
response function:

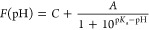
1.2biphasic
response function:

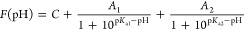
1.3(*F*, pH-dependent fluorescence intensity; *C* and *A*, fitted parameters).

Determination of switching quantum yields was carried out on a Cary 4000 UV–vis spectrophotometer by recording absorption spectra of RSFPs which had been illuminated for a defined period of time. Purified switchable fluorescent proteins were diluted to a 10 μM stock solution in 100 mM Tris pH 7.5 buffer and equilibrated overnight at 21 °C. The protein solutions were switched into respective on- and off-states (on-switching: 500 nm, 12.61 mW/cm^2^; off-switching: 405 nm, 6.02 mW/cm^2^), and absorption spectra of the respective state were recorded afterward. Protein solutions were switched into the off-state for 1, 2, 3, 4, 5, 10, 20, 30, and 60 s. On-switching was performed for 1, 2, 3, 4, 5, 10, 20, 30, 60, 120, and 180 s. A full spectrum was recorded after every switching step. Quantification of switching quantum yields was performed with custom-built MatLab routines taking concentration, optical path length, incident light intensity, extinction coefficient, and a photokinetic factor into account.^53^

### Fluorescence Lifetime

Fluorescence lifetimes were measured with a Quantaurus-Tau fluorescence lifetime spectrometer (Hamamatsu, Hamamatsu, Japan). Measurements were performed in quartz cuvettes with 470 nm excitation and detection at 516 nm. 10,000 photons were recorded after measurement of the internal response function with Polybead amino 0.10 μm microspheres (Polysciences, Warrington, PA, USA). Data were analyzed with version 3.0.0.80 of the Quantaurus-Tau software. The fluorescence lifetime of RSFPs was determined using a biexponential fit of the fluorescent decay curves. The displayed lifetime is an average of three different experiments.

### Chromophore Maturation

For measurement of oxygen-dependent chromophore maturation, an overnight culture of *E. coli* TOP10 cells transformed with the pBAD-Padron2 plasmid was used to inoculate 200 mL of LB medium containing ampicillin. After growth at 37 °C to an OD_595_ of 0.5, protein expression was induced with 0.02% arabinose and the culture was sealed to exclude oxygen. After 4 h incubation at 37 °C, cells were cooled on ice, pelleted by centrifugation, and opened up by several freeze–thaw cycles. After centrifugation, the Padron2 proteins were immediately purified from the supernatant within 20 min at 4 °C using His SpinTrap columns (GE Healthcare) using precooled buffers. The proteins were eluted with 20 mM sodium phosphate, 500 mM NaCl, and 30 mM imidazole pH 7.5. Fluorescence emission of the protein solution was measured every 2 min at 37 °C open to the air using a Cytation 3 plate reader (BioTek Instruments, Winooski, VT, USA) until the fluorescence reached a plateau.

### Switching Characterization

Switching kinetics and associated parameters were measured with the automated screening microscope in bacterial colonies. To ensure a maximal fluorescence signal, an autofocus was employed. Photobleaching was recorded with continuous 488 nm illumination at 2.3 ± 0.3 kW/cm^2^ with three to four averaged repetitions with 5–10 colonies each.

Residual fluorescence intensity in the ensemble off-state was quantified by switching proteins off with progressively increasing 405 nm illumination duration at different intensities with five colonies per condition in two to three repetitions. The residual fluorescence intensity in the ensemble off-state is defined as the remaining fluorescence of the protein ensemble when all or nearly all proteins are switched to the off-state; it is the reciprocal value of the achievable contrast between on- and off-states. Residual fluorescence intensities in the ensemble off-state were calculated from the consecutive on-switching curve. Graphs display averaged data with an ExpDec2 nonlinear curve fit calculated in OriginPro 2018b (OriginLab Corp., Northampton, MA, USA). Off-switching intensities are indicated in the respective graphs; on-switching was performed at 20.2 ± 0.9 kW/cm^2^. On-switching curves shown in Figure 3c were analyzed by switching proteins to 5% (Padron, Padron2) or 10% (Kohinoor) residual off-state fluorescence intensity with 405 nm and subsequent activation with 488 nm illumination at 1.3 kW/cm^2^. Three repetitions were measured with 10 colonies each. Measurements were averaged and normalized.

Switching fatigue was determined by repeatedly cycling the proteins between the ensemble on- and off-states using light doses that switched the proteins off to 5% (Padron, Padron2) or 10% (Kohinoor) residual fluorescence intensity and on to 95% of their maximal on-state fluorescence intensity. Off-switching 405 nm light intensity was 3.6 kW/cm^2^ and 488 nm intensity was 2.6 kW/cm^2^; three repetitions were measured with 10 colonies each. End points of the activation curves were used for illustration of the switching fatigue after normalization and averaging. All three RSFPs differed with regard to switching speed, and the light doses varied in switching fatigue measurements. Illumination duration and light doses are listed in Supporting Information Table S2. To estimate the influence of differing light doses, we repeated the measurements for Padron2 with Kohinoor illumination settings and *vice versa* (Supporting Information Figure S6).

Switching RSFPs into the off-state by using simultaneous illumination was recorded as follows: Proteins were initially subjected to a single full switching cycles with sequential illumination (10 ms of 405 nm illumination at 4.1 ± 0.1 kW/cm^2^; 50 ms of 488 nm illumination at 17.4 ± 0.3 kW/cm^2^). Subsequently, 10 cycles of switching to the off-state with simultaneous 405 and 488 nm illumination for 100 ms and switching to the on-state with 488 nm alone for 50 ms were recorded. For off-switching, different intensity combinations were measured (405 nm at 4.1 ± 0.1 kW/cm^2^ (low) or 56.2 ± 1.2 kW/cm^2^ (high); 488 nm at 1.1 ± 0.1 kW/cm^2^ (low) or 17.4 ± 0.3 kW/cm^2^ (high)); 488 nm illumination for the on-switching was at 17.4 ± 0.3 kW/cm^2^ in all measurements. For every intensity combination, three repetitions were measured with 10 colonies each. Off-switching curves shown in Supporting Information Figure S7 are averaged curves; graphs with 10 consecutive cycles in Supporting Information Figure S8 are representative single measurements.

For the evaluation of protein expression after growth at different temperatures, fluorescence intensities were probed with a 488 nm excitation intensity of 18.6 ± 0.5 kW/cm^2^ after growth at 37 °C for 24 h or 30 °C for 48 h.

Laser powers used were measured behind the objective lens (N PLAN L 20x/0.40, Leica Microsystems, Wetzlar, Germany) with a LabMax-TO laser power meter equipped with an LM-2 VIS sensor (Coherent, Santa Clara, CA, USA). For calculation of laser intensities, focal spot sizes were measured by focusing on a mirror surface on the microscope stage. Reflected light reached the detection path by usage of a 50/50 beam splitter, where a mirror was inserted and the spot was visualized with a SPC900NC webcam sensor (Philips, Amsterdam, Netherlands). Pixel size was calibrated with the image of a 10 μm scale, and intensity line profiles of the focal laser spots were measured. FWHM values were calculated, and a circular area with the FWHM as diameter was assumed for intensity calculations.

### Cloning

Mammalian expression vectors were created by cloning after PCR amplification of the Padron2 coding sequence with primers listed in Supporting Information Table S3.

pVimentin-Padron2: The coding sequence of Padron2 was inserted into pmKate2-vimentin (Evrogen, Moscow, Russia) with restriction enzymes AgeI and NotI, replacing mKate2. pKeratin-Padron2: The RSFP coding sequence of Padron2 was inserted into pTagRFP-keratin (Evrogen) with restriction enzymes *Kpn*I and NotI, replacing TagRFP.

Actin filaments were labeled indirectly with Padron2 fused to lifeact. The coding sequence was inserted into lifeact-EGFP pcDNA3.1(+)^54^ with enzymes *Bam*HI and NotI, replacing EGFP to create pLifeact-Padron2. Microtubules were indirectly labeled with a fusion construct of Map2 and Padron2 in pPadron2-Map2. For this, the coding sequence for Map2 was inserted into the backbone of pEGFP-Tub (BD Biosciences Clontech, Franklin Lakes, NJ, USA) with restriction enzymes XhoI and *Bam*HI after amplification from pDONR223-MAP2^55^ (forward primer, GATCTCGAGTGATGGCAGATGAACGGAAAGACGAAGC; reverse primer: GGTGGATCCTTATCACAAGCCCTGCTTAGCGAGTGCAGC), replacing the tubulin sequence. The mEGFP sequence was subsequently replaced by the one for Padron2 with NheI and *Bgl*II.

For expression in the endoplasmic reticulum, the Padron2 coding sequence was inserted into pEF/*myc*/ER (Invitrogen Life Technologies, Carlsbad, CA, USA) with enzymes *Sal*I and NotI to create pPadron2-ER, which added an N-terminal ER signal peptide and a C-terminal ER retention signal to the RSFP. Cytosolic expression was facilitated by expression of free Padron2 inserted into the TagRFP-N vector (Evrogen) with enzymes AgeI and NotI, replacing TagRFP to create pPadron2-N. Labeling of the nuclear pore complex was done with the expression plasmid pPadron2-Nup50 coding for a fusion construct. It was created by replacing the sequence of mEmerald with that of Padron2 with enzymes NheI and *Bgl*II in mEmerald-Nup50-C-10, which was a gift from Michael Davidson (Addgene plasmid no. 54209). Histones were labeled by fusing Padron2 to H2bn in pPadron2-Histon1 (H2bn). The EGFP sequence in pEGFP-Hist1H2BN^41^ was replaced with enzymes NheI and *Bgl*II. For mitochondrial localization, the DsRed coding sequence in pDsRed1-Mito (BD Biosciences Clontech) was replaced with that of Padron2 with enzymes AgeI and NotI creating pMito-Padron2. Centromeres were targeted with centromere protein C (CenpC) fusion constructs from plasmid pPadron2-CenpC1. The coding sequence for the target protein was PCR amplified from pDONR223-CenpC^40^ (forward primer, CAGATCTCGAGTGGCTGCGTCCGGTCTGGA; reverse primer, TCCGGTGGATCCTTAGCATTTCAGGCAACTCTCCT) and inserted into pEGFP-Tub (BD Biosciences Clontec) with enzymes XhoI and *Bam*HI replacing the tubulin sequence. The EGFP sequence was subsequently replaced with that of Padron2 with enzymes NheI and XhoI. Targeting of caveolae was facilitated by inserting the coding sequence for caveolin-1 after PCR amplification from pDONR223-CAV1^55^ (forward primer, TCCGCTAGCATGTCTGGGGGCAAAT; reverse primer, CCGGTGGATCCCGGGCCCGCGGTATTTCTTTCTGCAAGTTGATG) into the pPadron2-N with enzymes NheI and *Bam*HI creating pCaveolin1-Padron2. For peroxisomal targeting, Padron2 was inserted into pEGFP-PTS^56^ with enzymes NheI and *Bgl*II, replacing the EGFP sequence to create pPadron2-peroxi. This expression plasmid adds a C-terminal peroxisomal targeting sequence (PTS) to Padron2.

### Calculation of the Effective Point Spread Functions

The calculations of the effective PSFs are based on fast focus field calculations as described in ref (57). The simulations assumed the use of an oil immersion objective with 1.4 numerical aperture and a pinhole of 1 Airy unit (at 516 nm wavelength) as the optical system. The intensity profiles were calculated on the basis of the calculated spatial distribution of the fluorescent form of the protein, the excitation, and the confocal detection profile. In the case of the sequential illumination scheme, an activation pulse of 70 μs (7.6 μW) and an off-switching pulse of 350 μs (1.16 μW) were used to calculate the on-state population. The activation by the off-switching pulse was included in the simulations, whereas the activation by the excitation pulse (120 μs at 0.51 μW) was considered negligible. The population of the on-state at the different spatial positions was calculated for the simultaneous illumination scheme. We calculated the respective steady states by assuming light intensities at the back focal plane of the objective of 0.6 μW (488 nm) and 1.19 μW (405 nm).

### Microscopy

All images shown were recorded with transiently transfected HeLa cells 24 h post-transfection. Cells were mounted in DMEM without phenol red (Thermo Fisher) and imaged at ambient temperature with a customized 1C RESOLFT QUAD scanning microscope (Abberior Instruments, Göttingen, Germany). The microscope was equipped with an UPLSAPO 1.4 NA 100× oil immersion objective (Olympus, Shinjuku, Japan) as well as 405 and 488 nm continuous-wave lasers (both Cobolt, Solna, Sweden). The 405 nm doughnut-shaped beam was realized with an easy 3D module (Abberior Instruments). Fluorescence was detected with a SPCM-AQRH-13 photon counting module (Excelitas Technologies, Waltham, MA, USA) with a HC 550/88 detection filter. Laser powers were measured behind the objective with a PM200 power meter with the S170C sensor (ThorLabs, Newton, NJ, USA). The circular or ring-like area of both beams at FWHM intensity in the focus were determined and used for further calculations. Images and filament intensity line profiles measured with three adjacent lines were analyzed with the Fiji distribution of ImageJ (v1.52p)^58,59^ and OriginPro 2018b (OriginLab).

This manuscript has been previously submitted to the preprint server bioRxiv.^60^

## Supporting Information Available

(Figure S1) Protein sequence alignments; (Figure S2) mutated positions; (Figure S3) protein characteristics; (Figure S4) normalized pH absorption spectra; (Figure S5) chromophore maturation; (Figure S6) switching fatigue; (Figure S7) off-switching kinetics; (Figure S8) consecutive switching cycles; (Figure S9) off-switching performances; (Figure S10) simulated effective PSFs; (Figure S11) bleaching in RESOLFT imaging; (Table S1) extinction coefficients; (Table S2) applied light doses; (Table S3) amplification primers (PDF)
