# Supplementary material for: The Positive Switching Fluorescent Protein Padron2 Enables Live-Cell Reversible Saturable Optical Linear Fluorescence Transitions (RESOLFT) Nanoscopy without Sequential Illumination Steps
Source: ACS Nano. 2021 May 21;15(6):9509–21. doi: 10.1021/acsnano.0c08207 (PMC8291764; doi:10.1021/acsnano.0c08207)
Supplement: Supplementary file 1 — nn0c08207_si_001.pdf [file nn0c08207_si_001.pdf]

Supporting Information

The Positive Switching Fluorescent Protein Padron2 enables Live-Cell Reversible Saturable Optical Linear Fluorescence Transitions (RESOLFT) Nanoscopy without Sequential Illumination Steps

Timo Konen, Daniel Stumpf, Tim Grotjohann, Isabelle Jansen, Mariano Bossi, Michael Weber, Nickels Jensen, Stefan W. Hell, Stefan Jakobs

```
         •••••••••1••••••••10••••••••20••••••••30••••••••40••••••••50••••••••60••••••••70
Padron   ---------MSVIKPDMKIKLRMEGAVNGHPFAIEGVGLGKPFEGKQSMDLKVKEGGPLPFAYDILTMAFCYGNRVFAKY
Kohinoor ---------MSVIKPDMKIKLRMEGAVNGHPFAIEGVGLGKPFEGKQSMDLKVKEGGPLPFAYDILTMAFCYGNRVFAKY
Padron2  MVSKGEENNMAVIKPDMKIKLRMEGAVNGHPFAIEGVGLGKPFEGKQSVDLKVKEGGPLPFAYDILSMAFCYGNKVFIKY

         ••••••••80••••••••90••••••••100•••••••110•••••••120•••••••130•••••••140•••••••150
Padron   PENIVDYFKQSFPEGYSWERSMIYEDGGICNATNDITLDGDCYIYEIRFDGVNFPANGPVMQKRTVKWELSTEKLYVRDG
Kohinoor PENIVDYFKQSFPEGYSWERSMIYEDGGICIATNDITLDGDCYIYEIRFDGVNFPANGPVMQKRTVKWEPSTEKLYVRDG
Padron2  PENIVDYFKQLFPEGYSWERSMIYEDGGICNATNDITLDGDCFIYEIRFDGVNFPANGPVMQKRTVKWEPSTEKLYVRDG

         ••••••••160•••••••170•••••••180•••••••190•••••••200•••••••210•••••••220•••••••
Padron   VLKSDGNYALSLEGGGHYRCDFKTTYKAKKVVQLPDYHSVDHHIEIKSHDKDYSNVNLHEHAEAHSELPRQAK-----
Kohinoor VLKSDGNYALSLEGGGHYRCDSKTTYKAKKVVQLPDYHDVVHHIEIKSHDRDYSNVNLHEHAEAHSGLPRQAK-----
Padron2  VLKSDGNYALSLEGGGHYRCDSKTTYKAKKVVQLPDYHAVDHHIEIKSHDKDYSNVNLHEHAEAHSGLPGQAMDELYK
```

Figure S1. Protein sequence alignment of Padron, Padron2, and Kohinoor. Amino acids are numbered in accordance with the sequence of Dronpa. Mutations that were introduced to Dronpa to create the original Padron are highlighted in green, mutations of Padron2 and Kohinoor in comparison to Padron are highlighted in yellow.

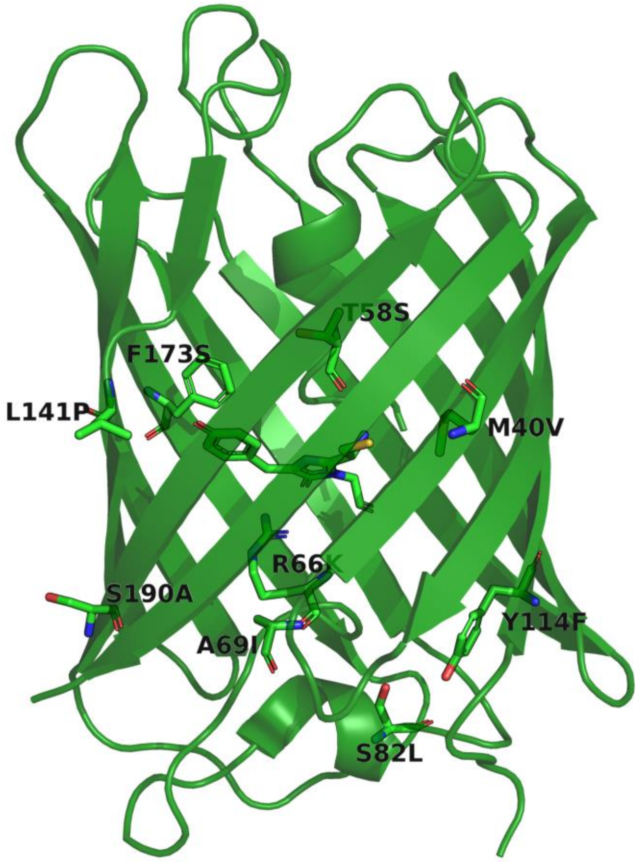

Figure S2. Mutated positions in Padron. Amino acids changed by mutagenesis during the generation of Padron2 are displayed in stick representation within the on-state structure of Padron (PDB ID: 3ZUJ), labels show mutations introduced in Padron2. R221 and E218 were not part of the solved crystal structure and are not highlighted. The structure was rendered with the PyMOL Molecular Graphics System, Version 2.3 Schrödinger, LLC.

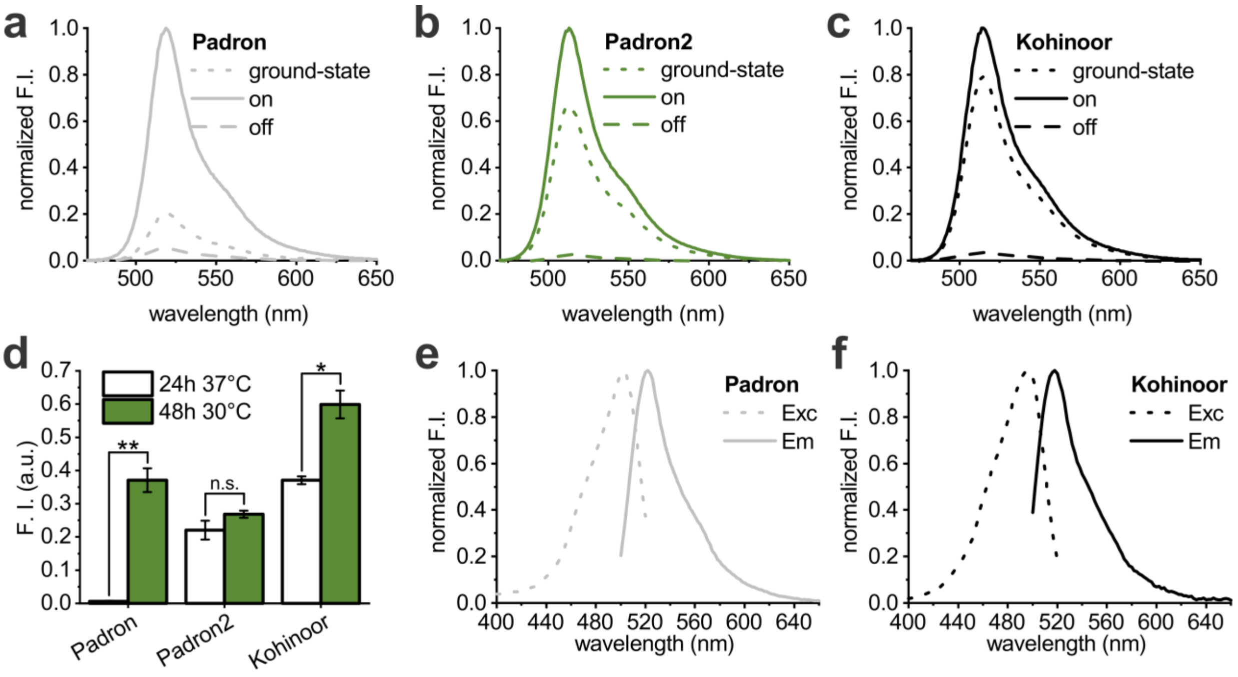

Figure S3. Protein characteristics. **(a, b, c)** Normalized fluorescence emission spectra of the ensemble on- and off-states as well as the equilibrated state of Padron (a), Padron2 (b), and Kohinoor (c). Excitation wavelength was 460 nm. **(d)** Absolute fluorescence intensity values in bacterial colonies used for Fig. 1g. Padron, Padron2, and Kohinoor fluorescence intensities were probed after growth at 37 °C for 24 h and growth at 30 °C for 48 h. p-values: ** = 2.2E-6, * = 9.8E-5, n.s. = 0.03. Two sample T-Tests calculated in OriginPro 2018b (OriginLab Corporation, Northhampton, MA, USA), equal variance assumed. **(e, f)** Normalized Excitation (Exc) and emission (Em) spectra for Padron (e) and Kohinoor (f). F.I., fluorescence intensity.

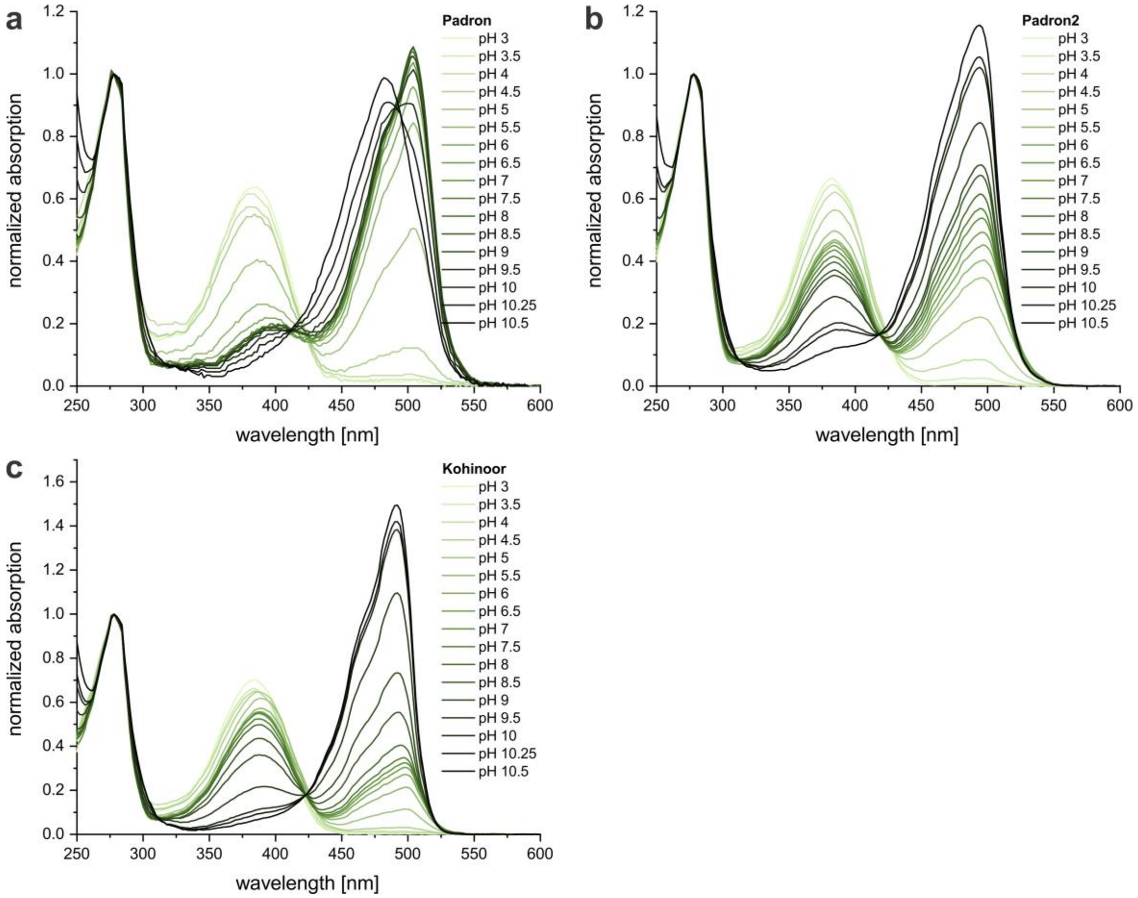

Figure S4. Normalized pH absorption spectra of **(a)** Padron, **(b)** Padron2, and **(c)** Kohinoor. Absorption spectra were measured in the equilibrated state at the pH-values indicated in the figure legends with a plate reader at room temperature and normalized to the absorption of aromatic side chains at 278 nm.

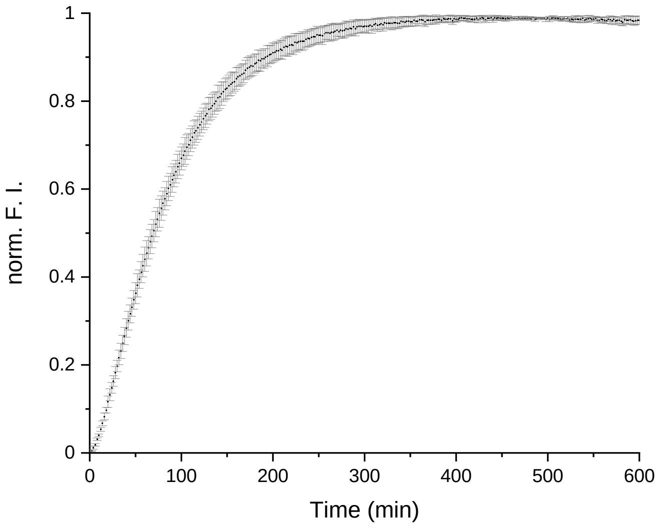

Figure S5. Chromophore maturation of Padron2 at 37 °C. The halftime of the chromophore maturation is ~70 minutes. Error bars represent the standard deviation of three independent experiments. F.I., fluorescence intensity.

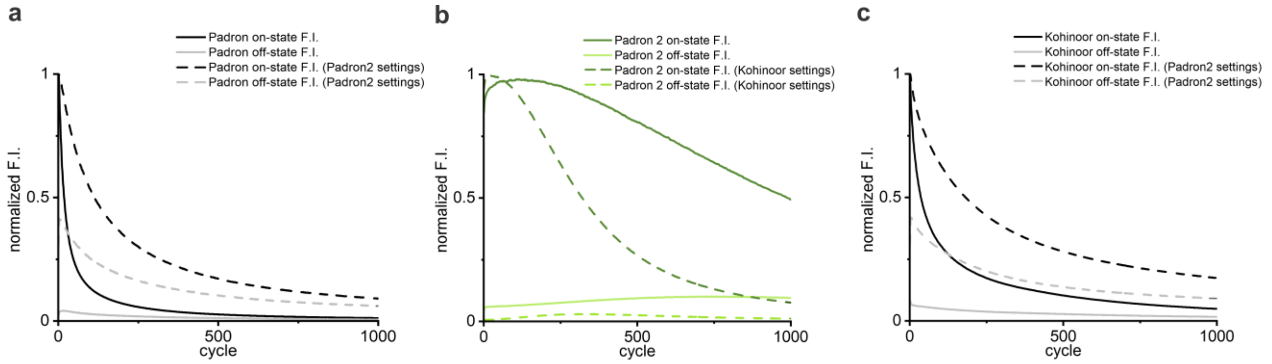

Figure S6. Switching fatigue of **(a)** Padron, **(b)** Padron2, and **(c)** Kohinoor. Graphs show switching fatigue as also shown in Fig. 3a (dark solid lines in a-c). The light solid lines (in a-c) show the residual fluorescence intensity values in the ensemble off-state. Solid lines show the fluorescence intensities with optimal light intensities and switching times for the respective protein and dashed lines show fluorescence intensities when switching with the light doses used for Padron2 and Kohinoor, respectively. Please note that in the first 50 switching cycles, the maximal fluorescence of Padron2 increases slightly (b), possibly suggesting light induced structural rearrangements in on-state Padron2. The underlying molecular mechanism is unknown. F.I., fluorescence intensity.

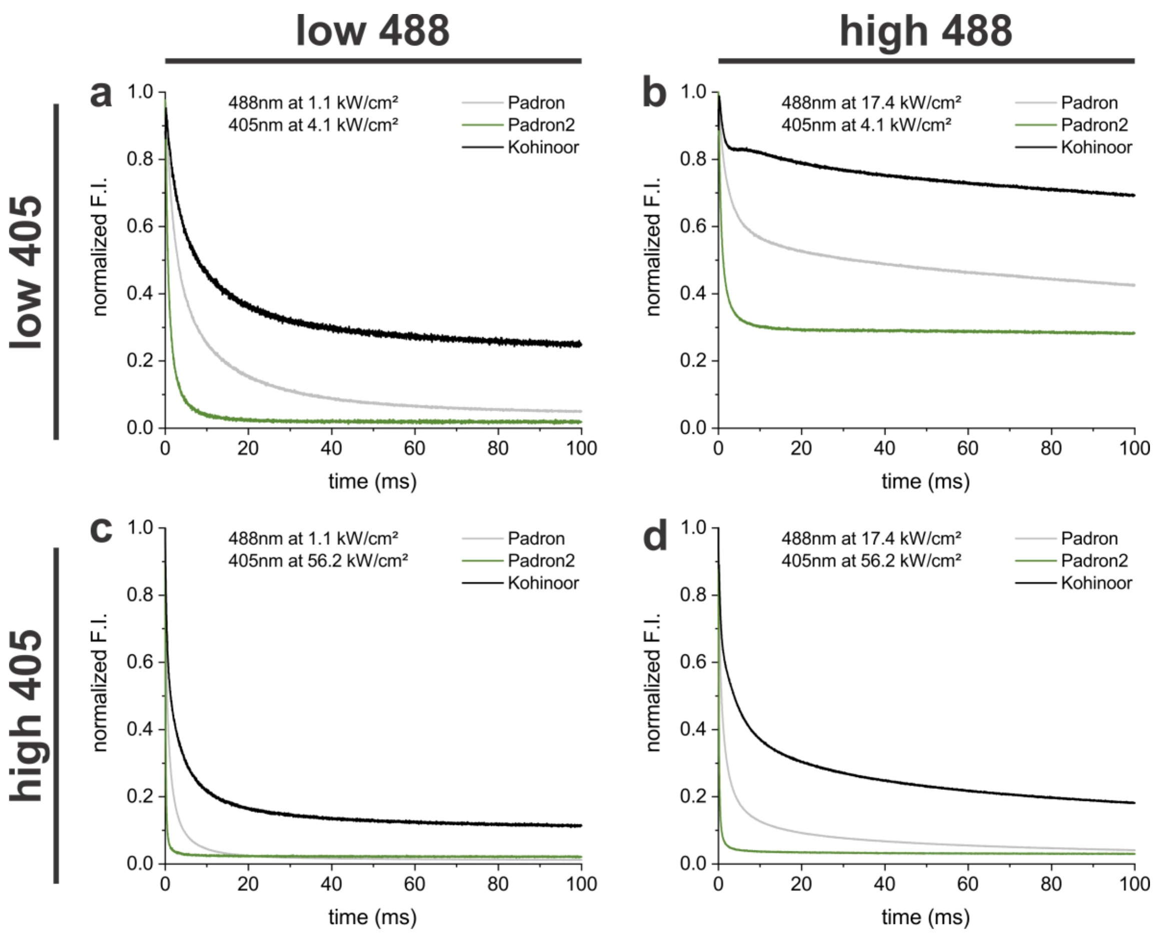

Figure S7. Off-switching kinetics with simultaneous 405 and 488 nm illumination. Fluorescence decay in bacterial colonies was measured at different intensity combinations of 405 nm (4.1 ± 0.1 kW/cm² (low) or 56.2 ± 1.2 kW/cm² (high)) and 488 nm (1.1 ± 0.1 kW/cm² (low) or 17.4 ± 0.3 kW/cm² (high)) illumination. **(a)** Low 405 nm and low 488 nm intensities, **(b)** low 405 nm and high 488 nm intensities, **(c)** high 405 nm and low 488 nm intensities **(d)** high 405 nm and high 488 nm intensities. Switching curves were normalized and are averaged data.

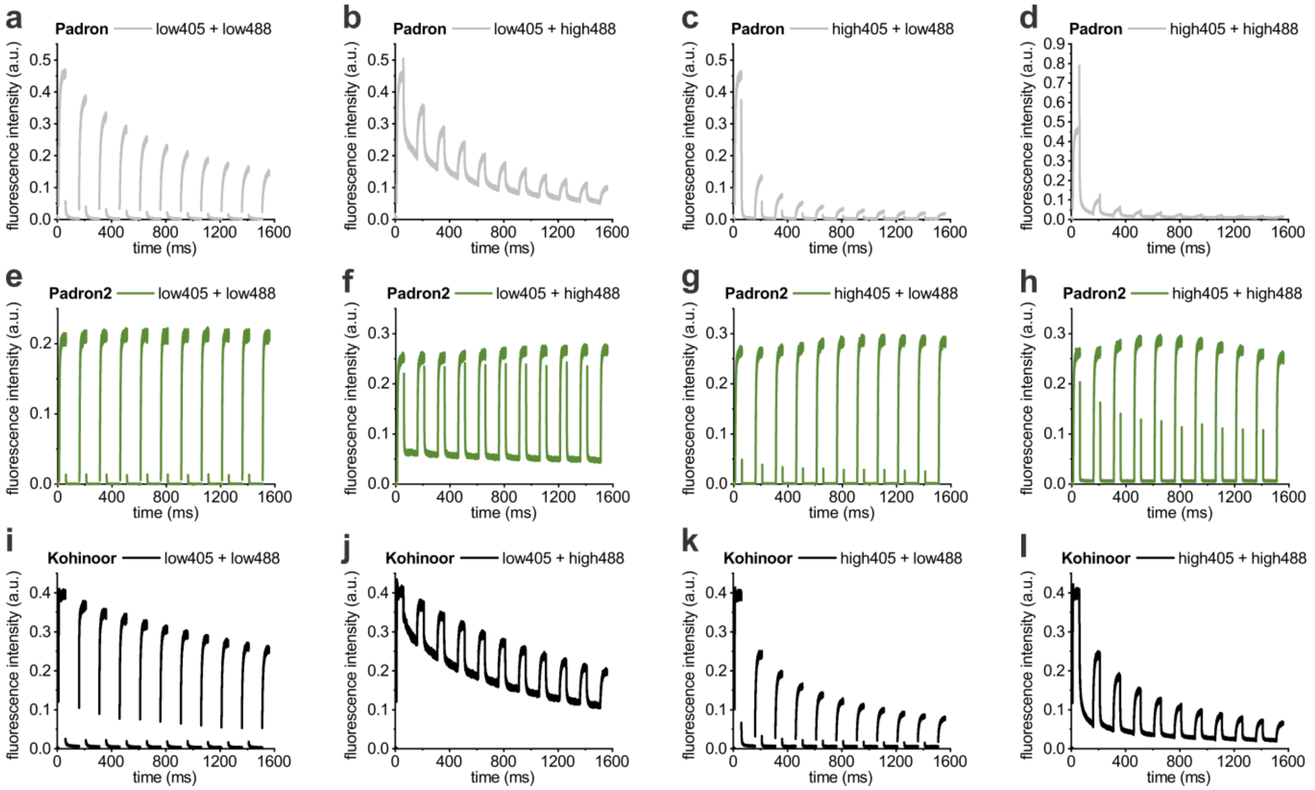

Figure S8. 10 consecutive switching cycles with different light intensities measured on bacterial colonies expressing the respective RSFPs. **(a-d)** Padron, **(e-h)** Padron2, and **(i-l)** Kohinoor. Proteins expressed in bacterial colonies were initially switched off and on again with 405 and 488 nm alone. For all experiments, the on- switching was done with light of 488 nm (50 ms of 17.4 ± 0.3 kW/cm²). For off-switching the samples were illuminated simultaneously with light of 405 and 488 nm with different intensities for 100 ms, as indicated in the figure. Following intensities were used: 405nm: 4.1 ± 0.1 kW/cm² (low) or 56.2 ± 1.2 kW/cm² (high); 488 nm: 1.1 ± 0.1 kW/cm² (low) or 17.4 ± 0.3 kW/cm² (high). Please note that the data points between the on- and the off-switching steps are not connected. The high 488 nm intensity used for off-switching is the same intensity as used for on-switching. As a result, in the off-switching curves of Padron and Kohinoor with high 488 nm no gap in the curves is visible (b, d, j, l). In the graphs with low 488 nm (a, c, e, g, i, k) switching, the on-switching and off-switching was performed applying different 488 nm intensities that are used for exciting fluorescence. Hence, a pronounced gap in the curves is visible. In the curves of Padron2 (f, h) a gap is visible, because of its fast off-switching. Graphs display representative measurements on a single colony.

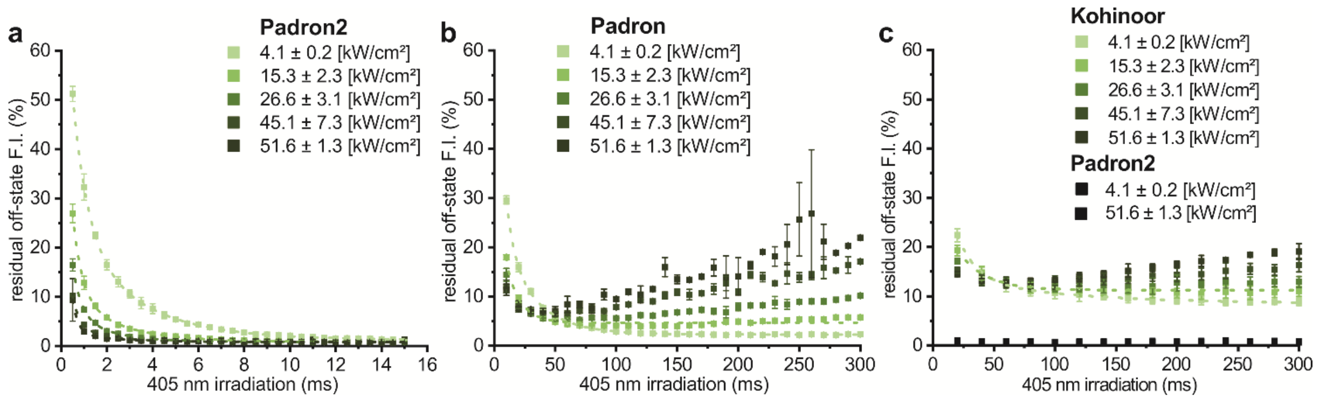

Figure S9. Off-switching performance of **(a)** Padron2, **(b)** Padron, and **(c)** Kohinoor. Data points are residual fluorescence intensities after different 405 nm illumination times (horizontal axes) at different intensities. Dashed lines represent exponential decay fitting data. Black squares in (c) are Padron2 data measured with the same illumination times used for Kohinoor for two different intensities, which resulted in similar residual fluorescence intensities (data points are shown for both intensities but are overlapping).

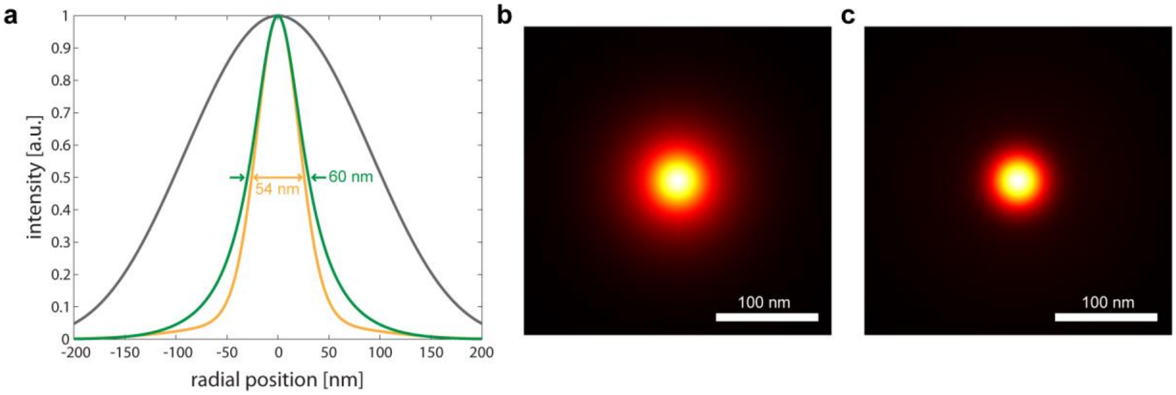

Figure S10. Simulated effective PSFs for Padron2 in sequential and simultaneous switching. **(a)** Central intensity profiles with FWHM of the effective PSFs of the sequential (orange) and simultaneous (green) switching scheme and excitation profile (gray). **(b)** 2D profile of the effective PSF of the simultaneous switching scheme. **(c)** 2D profile of the effective PSF of the sequential switching scheme. Scale bar: 100 nm.

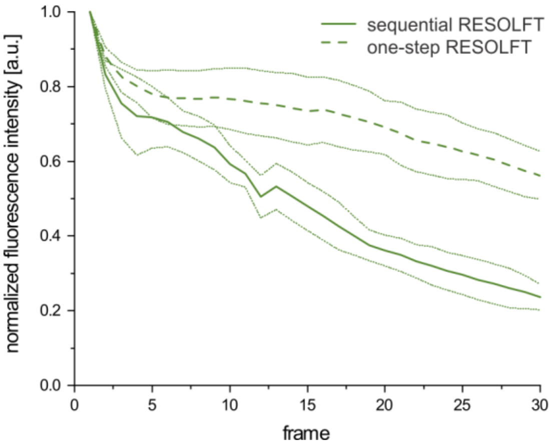

Figure S11. Bleaching in sequential and one-step RESOLFT (Reversible saturable optical linear (fluorescence) transitions) imaging. Hela cells were transiently transfected with vimentin-Padron2 expression plasmids and imaged after 24 h. 30 consecutive frames were recorded with sequential RESOLFT (activation, off-switching with a doughnut-shaped beam, and fluorescence readout) or one-step RESOLFT (simultaneous illumination with an off-switching doughnut-shaped beam and a Gaussian-shaped fluorescence readout beam). Total counts were measured in a central region of three time-lapse acquisitions recorded with identical settings in different cells and with comparable resolutions achieved. Total counts were normalized prior to averaging, standard deviations are indicated by the dotted lines adjacent to the graphs.

Table S1. Extinction coefficients of the on- and the off-states of Padron 2 and Kohinoor at 405 nm and 488 nm

	**Padron2**	**Kohinoor**
**Extinction coefficient on-state at 488 nm [$M^{-1}cm^{-1}$]**	12,700	11,150
**Extinction coefficient on-state at 405 nm [$M^{-1}cm^{-1}$]**	16,200	19,700
**Extinction coefficient off-state at 488 nm [$M^{-1}cm^{-1}$]**	45,300	38,000
**Extinction coefficient off-state at 405 nm [$M^{-1}cm^{-1}$]**	3,300	6,000

Table S2. Light doses applied for switching fatigue measurements.

	Illumination			
	405 nm<br>3.6 kW/cm^2	488 nm<br>2.6 kW/cm^2	**Total time**	**Total light dose**
**Padron**	60.8 ms	207 ms	267.8 ms	0.76 kJ/cm^2
**Padron2**	5.4 ms	73.1 ms	78.5 ms	0.21 kJ/cm^2
**Kohinoor**	164.5 ms	124.9 ms	289.4 ms	0.92 kJ/cm^2

Table S3. Primers used for amplification of the Padron2 coding sequence for cloning of mammalian expression plasmids.

Target structure/compartment	Primer orientation	Sequence
vimentin, mitochondria	fw	TCCACCGGTCGCCACCATGGTGAGCAAGGGCGAGGAG
vimentin, keratin, mitochrondria	rev	CCCTGCGGCCGCTTTACTTGTACAGCTCGTCCATGGC
keratin	fw	GACGGTACCGCGGGCCCGGGATCCACCGGTCGCCACCATGGTGAGCAAGGGCGAGGAG
lifeact	fw	AGGGGATCCACCGGTCGCCACCGTGAGCAAGGGCGAGGAGAACAAC
lifeact	rev	CGAGCGGCCGCTACTTGTACAGCTCGTCCATGG
Map2, CenpC	fw	GATCCGCTAGCGCTAATGGTGAGCAAGGGCGAGGAG
Map2, peroxisomes	rev	CACTCGAGATCTGAGTCCGGACTTGTACAGCTCGTCCATGGC
ER	fw	CTGCAGGTCGACATGGTGAGCAAGGGCGAGGA
ER	rev	TTCTGCGGCCGCCTTGTACAGCTCGTCCATGGCCTGCCCC
cytosolic, caveolin	fw	TCCACCGGTCGCCACCATGGTGAGCAAGGGCGAG
cytosolic, caveolin	rev	GTCGCGGCCGCTTACTTGTACAGCTCGTC
Nup50, histone H2bn	fw	TCCGCTAGCGCTACCGGTCGCCACCATGGTGAGCAAGGGCG
Nup50, histone H2bn	rev	CCACTCGAGATCTGAGTCCGGACTTGTACAGCTCGTCCATG
CenpC	rev	CACTCGAGATCTGAGTCCGGACTTGTACAGCTCGTCCATG
peroxisomes	fw	CGACGCTAGCATGGTGAGCAAGGGCG
